# Importance of extracellular vesicle secretion at the blood–cerebrospinal fluid interface in the pathogenesis of Alzheimer’s disease

**DOI:** 10.1186/s40478-021-01245-z

**Published:** 2021-08-23

**Authors:** Charysse Vandendriessche, Sriram Balusu, Caroline Van Cauwenberghe, Marjana Brkic, Marie Pauwels, Nele Plehiers, Arnout Bruggeman, Pieter Dujardin, Griet Van Imschoot, Elien Van Wonterghem, An Hendrix, Femke Baeke, Riet De Rycke, Kris Gevaert, Roosmarijn E. Vandenbroucke

**Affiliations:** 1 VIB Center for Inflammation Research, VIB, Ghent, Belgium; 2 Department of Biomedical Molecular Biology, Ghent University, Ghent, Belgium; 3 VIB Center for the Biology of Disease, VIB, Leuven, Belgium; 4 Department of Neurobiology, Institute for Biological Research, University of Belgrade, Belgrade, Republic of Serbia; 5 Department of Neurology, Ghent University Hospital, Ghent, Belgium; 6 Laboratory of Experimental Cancer Research, Department of Human Structure and Repair, Ghent University, Ghent, Belgium; 7 Cancer Research Institute Ghent, Ghent, Belgium; 8 VIB BioImaging Core, Ghent, Belgium; 9 VIB Center for Medical Biotechnology, VIB, Ghent, Belgium; 10 Department of Biomolecular Medicine, Ghent University, Ghent, Belgium

**Keywords:** Extracellular vesicles, Alzheimer’s disease, Blood–cerebrospinal fluid barrier, Choroid plexus, Complement

## Abstract

Increasing evidence indicates that extracellular vesicles (EVs) play an important role in the pathogenesis of Alzheimer’s disease (AD). We previously reported that the blood–cerebrospinal fluid (CSF) interface, formed by the choroid plexus epithelial (CPE) cells, releases an increased amount of EVs into the CSF in response to peripheral inflammation. Here, we studied the importance of CP-mediated EV release in AD pathogenesis. We observed increased EV levels in the CSF of young transgenic APP/PS1 mice which correlated with high amyloid beta (Aβ) CSF levels at this age. The intracerebroventricular (icv) injection of Aβ oligomers (AβO) in wild-type mice revealed a significant increase of EVs in the CSF, signifying that the presence of CSF-AβO is sufficient to induce increased EV secretion. Using in vivo, in vitro and ex vivo approaches, we identified the CP as a major source of the CSF-EVs. Interestingly, AβO-induced, CP-derived EVs induced pro-inflammatory effects in mixed cortical cultures. Proteome analysis of these EVs revealed the presence of several pro-inflammatory proteins, including the complement protein C3. Strikingly, inhibition of EV production using GW4869 resulted in protection against acute AβO-induced cognitive decline. Further research into the underlying mechanisms of this EV secretion might open up novel therapeutic strategies to impact the pathogenesis and progression of AD.

**Supplementary Information:**

The online version contains supplementary material available at 10.1186/s40478-021-01245-z.

## Introduction

Alzheimer’s disease (AD) is a progressive neurodegenerative disease mainly affecting the elderly population and accounting for 60–80% of dementia cases [1]. The major hallmarks of AD include the accumulation of extracellular amyloid beta (Aβ) plaques and the formation of neurofibrillary tangles, consisting of intracellular hyperphosphorylated tau (P-tau) protein. Although the exact pathogenic mechanism mediated by Aβ and tau proteins in AD is not clear, oligomeric species of these two proteins are believed to be responsible for the immunopathological changes in the brain. For instance, oligomeric species of Aβ were shown to be involved in the loss of brain barrier integrity, synaptic disruption, and neuronal cell death leading to severe cognitive impairment [2–7].

The barriers of the central nervous system (CNS), including the blood–brain barrier (BBB) and the blood–cerebrospinal fluid (CSF) barrier are responsible for the efflux of toxic molecules, including Aβ, from CSF to blood. The choroid plexus epithelial (CPE) cells form the blood–CSF barrier and provide an active interface between the CSF and the blood. Dysfunction of the brain barriers occurs in a number of neurological diseases, including AD [8, 9]. Further, chronological aging negatively influences the blood–CSF barrier. For instance, CPE cell height decreases by about 10–11% during life and cytoplasm is enriched with Biondi ring tangles and lipofuscin deposits, nuclei appear irregular and flattened and an increase in basement membrane thickness occurs [8, 10]. The pathophysiological changes in the choroid plexus (CP) lead to impaired clearing of Aβ from the CSF, thereby further aggravating the disease pathology. Accumulating evidence supports the notion that compromised blood–CSF barrier function and diminished clearance of Aβ from the CSF exacerbates AD pathology by elevating CSF Aβ levels [11–13]. Additionally, we reported that the intracerebroventricular (icv) injection of Aβ_1–42_ oligomers (AβO) induces inflammation in the CP as well as in the hippocampus and increases the expression of matrix metalloproteinases (MMPs) leading to blood–CSF barrier disruption [4]. Furthermore, we showed that tumor necrosis factor (TNF) signaling is induced in the CP of AD patients and AD mouse models, whereas TNF receptor 1 (TNFR1) abrogation reduced brain inflammation, blood–CSF barrier impairment and AβO-induced memory impairment [14].

Extracellular vesicles (EVs) are a large class of vesicles that originate from different subcellular compartments [15–18]. Based on their size and origin, EVs are classified into exosomes, microvesicles and apoptotic bodies [17]. EVs contain cytoplasmic signatures of the cells that they are derived from, which include proteins, miRNA, and long non-coding RNA. In the present decade, EVs evolved as important mediators of cellular communication and also as mediators for spreading pathogenic proteins in the CNS during neurodegenerative diseases [19, 20]. Several functions have been attributed to EVs in AD, for instance, some studies have shown that EVs have a role in Aβ removal [21–24] whereas other reports claim that EVs play a role in the formation of Aβ plaques [25]. However, it has been a matter of debate as to how pathogenic proteins such as Aβ and tau spread throughout the brain. Notably, both Aβ [26] and tau proteins [27, 28] were identified in EVs in the CSF of AD patients. Although the total amount of Aβ in the EVs was relatively low, the Aβ_42_/Aβ_40_ ratio was increased [26]. Also, elevated levels of tau and Aβ_1–42_ proteins in neuronal derived EVs in the blood of prodromal AD patients were reported [29, 30]. Interestingly, these plasma neuronal derived EVs seeded tau aggregation in vivo one month after they were injected into the hippocampus of wild-type (WT) mice [30]. In vitro toxicity to cerebral cortical neurons was shown for EVs isolated from CSF and plasma of sporadic late onset AD patients, plasma from APP/PS1 and 3xTg AD mice and culture medium from human neural cells expressing pathogenic presenilin 1 (PSEN1) [26]. Further, it was shown that EVs isolated from the human AD brain, more specifically the temporal neocortex, contain more AβO and that these EVs can be responsible for the neuron-to-neuron transfer of AβO in vitro [31]. Also, EVs isolated from the frontal cortex of AD patients contain significantly more pS369 tau and Aβ_1–42_ compared to EVs isolated from control brains [32]. EVs isolated from induced pluripotent stem cell neuronal cultures, either human tau-overexpressing [33, 34] or derived from a familial AD patient [35], seed tau pathology in the brain within five weeks after they were injected into the hippocampus of WT mice. Interestingly, neutral sphingomyelinase 2 (nSMase 2) inhibition using GW4869 or genetic deletion, resulting in blockage of EV formation and subsequent release from multivesicular bodies (MVBs), was shown to be beneficial in mitigating AD pathology in 5xFAD mice [36, 37] and reduced tau propagation in a mouse model of rapid tau propagation [38].

Next to Aβ and tau proteins, EVs also contain several additional proteins that might be altered in the course of AD pathology [39]. For instance, it was shown that astrocyte-derived EVs from plasma of AD patients and patients with mild cognitive impairment (MCI) who are converting to dementia contain significantly higher complement levels compared to controls and stable MCI patients [40, 41]. Furthermore, astrocyte-derived EVs separated from plasma of AD patients exert neurotoxicity in neuronal cultures due to their high levels of several complement proteins [42]. The complement system is a rapid and efficient immune surveillance system, but when imbalanced it contributes to a variety of disorders including AD [43, 44]. Several complement proteins including complement component 1q (C1q) and complement component 3 (C3) were detected in the hippocampus, the temporal cortex and the frontal cortex of AD patients and were often co-localizing with Aβ plaques [45–52]. A meta-analysis of multiple studies showed significantly higher C3 concentrations in the CSF of AD patients compared to controls [53]. In the hippocampus of pre-plaque J20 transgenic mice, pre-plaque APP/PS1 transgenic mice and WT mice that received an icv injection of AβO, synaptic C1q and C3 were elevated [54]. Strikingly, inhibition of C1q, C3 or the microglial complement receptor CR3 reduces the extent of synapse loss in these AD models, suggesting that microglia eliminate synapses through a complement-dependent mechanism in AD models prior to plaque formation [54]. In aged APP/PS1 mice, genetic C3 deficiency reduced plaque-related synapse and neuron loss in the hippocampus and protected against cognitive decline, despite an increased plaque burden [55]. Moreover, tau pathology has also been linked to complement activation since tau P301S mice show robust complement protein expression in the brain whereas genetic C3 deficiency in this model reduces neuron loss and brain atrophy, partially normalizing neurophysiological and behavioral alterations [52].

Previously, we reported increased EV secretion into the CSF by CPE cells upon inflammatory stimuli [56]. Here, we investigated the role of CP-derived EVs in AD pathogenesis. We could show that in response to AβO, the CP secretes EVs that contain several inflammatory proteins, including C3, into the CSF. These CP-derived, AβO-induced EVs evoke a pro-inflammatory response in brain target cells whereas inhibition of EV production using GW4869 protects against the AβO-induced cognitive decline.

## Materials and methods

### Mice

For the experiments with icv injections of AβO, C57BL/6J mice were housed in a specific pathogen-free (SPF) animal facility. Heterozygous B6-Tg (Thy1-APPswe; Thy1-PSEN1 L166P) mice in a C57BL/6J background, further referred to as APP/PS1 mice, and corresponding non-transgenic littermates (referred to as WT mice) were bred in a conventional animal facility. These mice harbor human transgenes for both the amyloid precursor protein (APP) bearing the Swedish mutation (KM670/671NL) and presenilin 1 (PSEN1) bearing an L166P mutation, both under the control of the neuron-specific Thy1 promotor [57]. All animals were housed in groups of 4–6 per cage with ad libitum access to food and water and a 14 h light/10 h dark cycle. Both male and female mice were used, except for the cognition experiments where we solely worked with female mice. C57BL/6J mice were between 8 and 10 weeks of age and APP/PS1 mice were between 7 and 40 weeks of age at the time of the experiments. All experiments were officially approved by the ethical committee of the Faculty of Sciences, Ghent University.

### Preparation of Aβ_1–42_ oligomers

AβO were prepared as described previously [4, 14, 58, 59]. In brief, Aβ_1–42_ (A-1163-1; rPeptide) or scrambled peptide (A-1004-1; rPeptide) was dissolved in hexafluoroisopropanol (HFIP; 105228; Sigma-Aldrich) at 1 mg/ml. Next, HFIP was removed in a SpeedVac vacuum concentrator. The resulting peptides were dissolved in DMSO and purified using a 5 ml HiTrap desalting column (17-408-01; GE Healthcare). The monomeric Aβ_1–42_ was eluted with Tris–EDTA buffer (50 mM Tris and 1 mM EDTA, pH 7.5). The eluted peptide concentration was measured using Pierce BCA Protein Assay (23225; Thermo Scientific) according to the manufacturer’s instructions. The resulting peptides were allowed to oligomerize at room temperature (RT) for 2 h, followed by dilution to 1 µg/ml in PBS. All the icv injections were performed within 2 h after the oligomerization step.

### Intracerebroventricular injection

For icv injections, 8–10 weeks old C57BL/6J mice were anesthetized with isoflurane and mounted on a stereotactic frame. A constant body temperature of 37 °C was maintained using a heating pad. Injection coordinates were measured relative to the bregma intersection (anteroposterior 0.07 cm, mediolateral 0.1 cm, dorsoventral 0.2 cm) and were determined using the Franklin and Paxinos mouse brain atlas. By using a Hamilton needle, 5 µl of either scrambled peptide (1 µg/ml) or AβO (1 µg/ml) was injected into the left lateral ventricle. For co-injections, 2.5 µl (2 µg/ml) of either scrambled peptide or AβO was mixed with either 2.5 µl of GW4869 (4.3 mM in DMSO; D1692; Sigma) or vehicle (DMSO).

### Cerebrospinal fluid isolation

CSF was collected using the cisterna magna puncture method as described earlier [4, 14, 60]. Briefly, capillaries to isolate CSF were made from borosilicate glass capillary tubes (B100-75-15; Sutter Instruments) using the Sutter P-87 flaming micropipette puller (pressure 330 Pa, heat index 300). Just before CSF isolation, mice were anesthetized with 200 µl of ketamine/xylazine (100 mg/kg ketamine; 20 mg/kg xylazine). An incision was made inferior to the occiput and disinfected with 70% ethanol. The dura mater was exposed by separating the muscle tissue on the dorsal side of the skull. Next, the animal was mounted at an angle of 135° and CSF was collected by puncturing the dura mater of the cisterna magna using the capillary needle. On average, we were able to isolate 5 µl of CSF per mouse.

### Tissue sample isolation

For RNA analysis, mice were transcardially perfused with a mixture of D-PBS (Sigma), 0.002% heparin sodium (5000 IU/ml; Wockhardt) and 0.5% bromophenol blue (Sigma). Next, CP was dissected from the brain tissue. Isolated CP samples were snap frozen in liquid nitrogen for RNA analysis. For immunohistochemical analysis, mice were transcardially perfused with 4% paraformaldehyde (PFA) after which brain samples were post-fixed in 4% PFA overnight (ON) followed by paraffin embedding.

### qPCR

RNA was isolated from the CP and the mixed cortical cultures (MCC) using the Aurum Total RNA Mini Kit (732-6820; Bio-Rad). RNA concentration was measured using the Nanodrop 1000 (Thermo Scientific) and cDNA was prepared using an iScript cDNA synthesis kit (172-5038; Bio-Rad). Real-time qPCR was performed with the Light Cycler 480 system (Roche) using the SensiFAST SYBR No-ROX Kit (BIO-98002; Bioline). Expression levels of the genes of interest were normalized to two or three stably expressed reference genes, as determined by the geNorm Housekeeping Gene Selection Software [61]. *Hprt*, *Rpl* and *Ubc* were used as the reference genes for CP and MCC. The expression data are displayed as relative expression normalized to the lowest expressed sample, unless mentioned differently. The primer sequences of the forward and reverse primers for the different genes are provided in Additional file 1: Appendix Table S1.

### Immunohistochemistry

For immunostainings on mouse brain sections, 5 µm sections were prepared from the paraffin embedded samples. After paraffin removal, samples were treated with citrate buffer (S2031; DAKO) followed by blocking with 5% BSA after washing with PBS. Next, sections were incubated ON with the primary antibodies anti-Alix (1:1000; ab76608; abcam), anti-Flotillin1 (FLOT1) (1:600; ab41927; abcam), anti-CD63 (1:200; sc-31214; Santa Cruz biotechnology), anti-RAB5 (1:500; ab18211; Abcam), anti-AnnexinA2 (ANXA2) (1:200; ab54771; Abcam) and anti-C3 (1:100; PA5-21349; Thermo Scientific). After a washing step, sections were incubated with the secondary antibodies goat anti-rabbit biotin (1:500; E0432; DAKO) or goat anti-mouse biotin (1:500; E0433; DAKO) for 2 h at RT. Next, amplification of the signal was performed using the ABC system (PK-6100; Vector laboratories) and TSA (SAT700001EA; Perkin Elmer) according to manufacturer’s instructions and samples were incubated with streptavidin-DyLight 633. Finally, the samples were counterstained with Hoechst (1 µg/ml) and the sections were mounted using 2% n-propyl gallate. A Leica TCS SP5 II confocal microscope or a Zeiss LSM780 confocal microscope was used for imaging. 3D reconstructions of z-stacks were created with Volocity.

### Transmission electron microscopy on CP tissue

The CP of the fourth ventricle was dissected from 10 weeks old APP/PS1 or non-transgenic littermates and from C57BL/6J mice 6 h after the icv injection of either AβO or scrambled peptide. Next, samples were prepared for transmission electron microscopy (TEM) as we described previously [14]. After isolation, the CP was fixed in a solution of 2.5% glutaraldehyde and 4% formaldehyde dissolved in 0.1 M sodium cacodylate buffer, pH 7.2 for 4 h at RT followed by ON fixation at 4 °C. After washing in buffer, the samples were post-fixed in 1% OsO_4_ with 1.5% K_3_Fe(CN)_6_ in 0.1 M sodium cacodylate buffer at RT for 1 h. Next, the samples were washed in ddH_2_O and subsequently dehydrated through a graded ethanol series, including a bulk staining with 1% uranyl acetate at the 50% ethanol step followed by embedding in Spurr’s resin.

Ultrathin sections of a gold interference color were cut using an ultramicrotome (Leica EM UC6), followed by a post-staining in a Leica EM AC20 for 40 min in uranyl acetate at 20 °C and for 10 min in lead stain at 20 °C. Sections were collected on formvar-coated copper slot grids. Grids were viewed with a JEM 1400plus TEM (JEOL, Tokyo, Japan) operating at 60 kV. To quantify the amount of MVBs and intraluminal vesicles (ILVs) in the samples, two different regions of 20 CPE cells were manually counted for each biological replicate. Consequently, the average amount of MVBs and ILVs in 40 CPE cells per cell section was obtained.

### CP explant cultures

CP explant cultures were prepared as we described previously [56]. Three hours after the icv injection with (1) AβO or scrambled peptide or (2) scrambled peptide + vehicle, AβO + vehicle or AβO + GW4869, anesthetized C57BL/6J mice were transcardially perfused with serum free DMEM-F12 medium (11320074; Gibco) after which the CP tissue was dissected. For the qEV quality control experiment, a similar approach with non-injected, C57BL/6J mice was used. The explants were incubated in 24-well plates containing 350 µl of Opti-MEM (11058-021; Gibco) medium (supplemented with penicillin/streptomycin (pen/strep; P4333; Sigma); non-essential amino-acids (NEAA; M-7145; Sigma), sodium pyruvate (S8636; Sigma) and L-glutamine (BE17-605F; Lonza)) per well for 16 h at 37 °C and 5% CO_2_. Conditioned medium was collected and briefly centrifuged at 300 *g* for 5 min and 4 °C to eliminate cellular debris. For the qEV quality control experiment, this conditioned medium was subjected to qEV separation (see below). For the AβO experiments, at this point a part of the supernatant was stored for Nanoparticle Tracking Analysis (NTA) and cytokine/chemokine analysis using the Bio-Plex cytokine assays kit (Bio-Rad) according to the manufacturer’s instructions. The remaining supernatant (300 µl) was either directly transferred onto the MCC or immediately (without intermediate storage) subjected to qEV separation whereafter the EVs were transferred to the MCC. Explant secretome derived from the CP of one mouse was used to stimulate one well of MCC.

### Mixed cortical cultures

MCC were prepared as we described previously [56]. Briefly, the cerebral cortex and hippocampus were obtained from 3 to 5 neonatal P1/P2 C57BL/6J mice from which the meninges were completely removed. Tissues were finely minced with a surgical scalpel in cold PBS. Next, samples were spun at 300 g for 5 min after which they were digested with 0.25% trypsin (T-4424; Sigma) for 15 min in a water bath at 37 °C. Cells were again centrifuged at 300 *g* for 5 min and washed twice with ice-cold PBS. Typically, 3–5 × 10^5–6^ cells were obtained from each pup. Cells were resuspended in DMEM (41965-062; Gibco) culture medium (supplemented with 10% fetal bovine serum (FBS); pen/strep (P4333; Sigma); NEAA (NEAA; M-7145; Sigma), sodium pyruvate (S8636; Sigma) and L-glutamine (BE17-605F; Lonza)) and plated directly in 24-well plates coated with 0.1% poly-L-lysine (P6282, Sigma). Cells were maintained in standard tissue culture conditions and 50% of the medium was replaced every two days. The presence of three different cell types was confirmed by staining GFAP, IBA1 and TUBB3 for astrocytes, microglia and neurons, respectively. When the cells reached confluence, they were incubated for 24 h at 37 °C and 5% CO_2_ with either the complete supernatant or qEV enriched EVs separated from the supernatant of CP explants derived from scrambled or AβO injected mice. Next, conditioned medium was collected and stored at − 80 °C until cytokine/chemokine analysis by using the Bio-Plex cytokine assays kit (Bio-Rad) according to the manufacturer’s instructions. TRIzol reagent (250 µl/well; 15596018; Gibco) was added to the cells whereafter they were stored at − 20 °C until RNA isolation and qPCR analysis.

### Primary cultures

Primary mouse CPE cultures were prepared as we described previously [56]. In brief, 2–7 day old C57BL/6J pups were decapitated and CP was isolated under a dissection microscope. After washing with HBSS, enzymatic dissociation of the CP was achieved using pronase (53702; Sigma) for 5–7 min at 37 °C. Digestion was stopped by adding an excess of HBSS buffer, after which the cells were washed twice with HBSS. The cell pellet was resuspended in DMEM-F12 (11320074; Gibco) culture medium (supplemented with 10% FBS; pen/strep (P4333; Sigma); NEAA (M-7145; Sigma), sodium pyruvate (S8636; Sigma) and L-glutamine (BE17-605F; Lonza)) and plated on laminin (L2020; Sigma) coated transwell inserts. For the proteomics experiment ~ 10 pups per 75 mm transwell dish (3419; Corning), for the NTA experiment ~ 10 pups per 6-transwell plate (CLS3412-24EA; Corning) and for the qEV quality control experiments ~ 10 pups per standard 100 mm dish were used. Two days after plating, culture media were replaced with DMEM-F12 containing cytosine arabinoside (Ara-C; C1768; Sigma) to prevent fibroblast growth. Cultures were maintained at 37 °C in 5% CO_2_. When the cells reached confluence, the culture medium was replaced by Opti-MEM (11058-021; Gibco) medium (supplemented with pen/strep (P4333; Sigma); NEAA (NEAA; M-7145; Sigma), sodium pyruvate (S8636; Sigma) and L-glutamine (BE17-605F; Lonza)) 4 h prior to the stimulus. The cells were stimulated with 5 µg/ml AβO or scrambled peptide on the apical side for 2 h, followed by washing with HBSS and addition of Opti-MEM. Conditioned medium was collected from the apical side of the transwells after 24 h of incubation at 37 °C and 5% CO_2_ and subjected to NTA or qEV separation for proteomics. For the qEV quality control experiment, conditioned medium was serially collected from non-stimulated cells with 48 h time-intervals and a maximum of six collections.

### EV separation from CP explant cultures using qEV

Conditioned medium of CP explant cultures (350 µl per CP explant) obtained from AβO or scrambled peptide icv injected mice or non-injected mice was collected after 16 h of incubation at 37 °C and 5% CO_2_. Next, the conditioned medium was centrifuged at 300 *g* for 5 min at 4 °C to remove debris. For the qEV quality control experiment, the conditioned medium of several CP explants derived from non-injected mice was pooled and concentrated using the Amicon Ultra-4 Centrifugal Filter Unit with Ultracel-10 membrane (UFC801024; Amicon). Next, the supernatant was diluted in PBS to obtain a final volume of 500 µl, after which the EVs were immediately (without intermediate storage) separated using the qEV original columns (qEV original/70 nm SEC columns; Izon Science) according to the manufacturer’s instructions. After discarding the void volume (3 ml), the EVs were eluted in PBS in 500 µl fractions. For the qEV quality control experiment, fractions 1 to 20 were collected using the Automatic Fraction Collector (Izon Science) and stored ON at 4 °C after which they were subjected to ZetaView analysis and protein concentration measurement. After storage at − 20 °C, fractions 2 and 3 were analyzed by ExoView analysis, negative staining and western blot. For the AβO experiment, fractions 2 and 3 were collected, pooled and concentrated to approximately 30 µl using the Amicon Ultra-0.5 Centrifugal Filter Unit with Ultracel-10 membrane (UFC501024; Amicon). Next, Opti-MEM was added to a final volume of 300 µl that was transferred onto the MCC.

### EV separation from primary cultures using qEV

Conditioned medium (5 ml per transwell) from CPE primary cells stimulated with 5 µg/ml AβO or scrambled peptide was collected from the apical side of transwell inserts after 24 h of incubation at 37 °C and 5% CO_2_. For the qEV quality control experiment, conditioned medium (10 ml per dish) was serially collected from non-stimulated cells with 48 h time-intervals and a maximum of six collections. The conditioned medium was centrifuged at 300 *g* for 5 min at 4 °C to remove debris, filtered through a 0.22 µm filter and concentrated to 200–300 µl using an Amicon Ultra-15 Centrifugal Filter Unit with Ultracel-100 membrane (UFC910024; Amicon). For the qEV quality control experiment, serially collected samples were stored at − 80 °C at this stage until all samples could be pooled to a final volume of ~ 20 ml, after which they were concentrated again to 200–300 µl using the same type of Amicon filter. Next, the supernatant was diluted in PBS to obtain a final volume of 500 µl, after which the EVs were either immediately (without intermediate storage; proteomics experiment) or after storage at − 80 °C (qEV quality control experiment) separated using the qEV original columns (qEV original/70 nm SEC columns; Izon Science) according to the manufacturer’s instructions. After discarding the void volume (3 ml), the EVs were eluted in PBS in 500 µl fractions. For the qEV quality control experiment, fractions 1 to 20 were collected using the Automatic Fraction Collector (Izon Science) and stored ON at 4 °C after which they were subjected to NanoSight analysis and protein concentration measurement. After storage at − 20 °C, fractions 2 and 3 were analyzed by ExoView analysis, negative staining and western blot. For the proteomics experiment, fractions 2 and 3 were collected, pooled, vacuum evaporated and lysed in RIPA buffer.

### Transmission electron microscopy on EVs

To verify the separation of qEV enriched EVs, we performed negative staining on qEV fractions of CP explant cultures and CPE primary cultures obtained as described above. For the visualization of EVs, 20 µl of sample was spotted on a parafilm sheet. Formvar/C-coated hexagonal copper grids (EMS G200H-Cu), which were glow discharged for 10 s, were placed on top of the droplet for 1 min with the coated side of the grid down. The grids were washed 5 times in droplets of Milli-Q water, stained with 1% (w/v) uranyl acetate for 10 s and air dried for 24 h before imaging. Visualization of the samples was done using a JEM 1400plus TEM (JEOL, Tokyo, Japan) operating at 80 kV.

### Nanoparticle Tracking Analysis using NanoSight

To determine the amount of particles in (1) CSF of APP/PS1 mice or non-transgenic littermates and C57BL/6J mice icv injected with either AβO or scrambled peptide, (2) medium of primary CPE cells stimulated with AβO or scrambled peptide, (3) medium of CP explants derived from AβO or scrambled peptide injected mice and (4) EV fractions separated as described above from medium of primary CPE cultures, the samples were centrifuged at 300 *g* for 5 min at 4 °C after their collection in order to remove debris. The supernatant was stored ON at 4 °C prior to the analysis. Just before the measurement, the samples were diluted in PBS until their particle concentration was within the optimal concentration range of the NTA software (3 × 10^8^–1 × 10^9^). Next, they were injected into the NanoSight LM10-HS instrument (Malvern, UK) equipped with a 405 nm laser. Three 60 s videos were recorded for each sample with camera level and detection threshold respectively set at 11 and 3. Temperature was monitored throughout measurements. The videos were analyzed with NTA software version 2.3 to determine the concentration and size of measured particles. NTA post-acquisition settings were optimized and kept constant between samples. Absolute numbers were recorded and back-calculated using the dilution factor. The NanoSight system was calibrated with polystyrene latex microbeads at 50, 100, and 200 nm (Thermo Scientific) prior to analysis.

### Nanoparticle Tracking Analysis using ZetaView

To determine the amount of particles in qEV enriched fractions of CP explant cultures, a ZetaView analysis was performed. The samples were collected as described above and stored ON at 4 °C in low adhesion tubes prior to the analysis. Just before the measurement, the samples were diluted in PBS until their particle concentration was within the optimal concentration range (100–400 particles/frame) of the instrument. Next, they were injected into the ZetaView PMX-120 instrument (Particle Metrix, Germany) equipped with a 520 nm laser. For each measurement, two cycles of each 11 positions were performed, capturing 30 frames per position. The settings were kept constant for all samples (focus: autofocus; camera sensitivity: 82; shutter: 100; scattering intensity: detected automatically; temperature: 23 °C; video length: high). The videos were analyzed by the ZetaView software version 8.04.02 SP2 to determine the concentration and size of measured particles. NTA post-acquisition settings were optimized and kept constant between samples (min size: 10; max size: 1000; min bright: 30; tracelength: 15). Absolute numbers were recorded and back-calculated using the dilution factor. The ZetaView system was calibrated with polystyrene latex microbeads at 100 nm (Particle Metrix) prior to analysis.

### ExoView analysis

To determine the amount of CD9 and CD81 positive EVs in CSF isolated from C57BL/6J mice 6 h after the icv injection of either AβO or scrambled peptide and in fractions 2 and 3 of qEV enriched EVs from CP explant medium and CPE primary cultures, we applied the ExoView technology using the ExoView Mouse Tetraspanin Kit (EV-TETRA-M; NanoView Biosciences) and the ExoView R100 reader (NanoView Biosciences, Boston, USA). After their isolation, the CSF samples were centrifuged at 300 *g* for 5 min at 4 °C in order to remove debris and stored at − 80 °C. The qEV enriched EVs from CP explant medium and primary CPE cells were stored at − 20 °C in low adhesion tubes prior to the analysis which was performed following the manufacturer’s guidelines. In brief, the CSF samples and qEV enriched fractions from CP explant medium and primary CPE cells were diluted respectively 1:25, 1:100 and 1:10 using incubation solution whereafter 35 µl sample was directly incubated on the chip (coated with CD9, CD81 and IgG control antibodies) for 16 h at RT without agitation in a sealed 24-well plate. For the standard protocol, chips were washed three times and subjected to staining using anti-CD81 Alexa-555 (1:2000 in blocking solution) for 1 h at RT without agitation. For the transthyretin (TTR) detection, an additional fixation and permeabilization step (10 min each) were included before staining with anti-CD81 Alexa-555 (1:2000 in blocking solution) and TTR Alexa-647 (ITA7301; G-Biosciences; 1:100 in blocking solution) for 1 h at RT without agitation. After incubation, chips were washed three times in wash solution, once in rinse solution and subsequently dried and scanned. The data obtained were analyzed using the NanoViewer analysis software version 2.8.10.

### Protein analysis

Protein concentrations of qEV enriched EVs were measured using the Micro BCA Protein Assay kit (23235; Thermo Scientific) according to the manufacturer’s instructions. Both the samples and the standard were diluted 1:1 with 0.4% SDS prior to the measurement.

### Western blot analysis

CP explant tissue and primary CPE cells were homogenized in Tris buffered saline (50 mM Tris pH 7.5, 150 mM NaCl, 1% Triton X-100 in PBS) supplemented with protease inhibitors (Roche). Protein concentration was measured using the Pierce BCA protein assay (23225; Thermo Scientific). An equal loading amount of 8 µg cell lysate or qEV enriched EVs separated from CP explant medium and CPE primary cultures was mixed with sample buffer (0.35 M Tris HCl pH 6.8, 10% sodium dodecyl sulphate (SDS), 35% glycerol, 5% β-mercaptoethanol, 0.5% bromophenol blue) and boiled for 10 min at 95 °C. Proteins were separated on a 10% SDS-PAGE gel and transferred to a 0.45 µm nitrocellulose membrane via wet blotting. The nitrocellulose membrane was blocked for 1 h at RT using 1:2 diluted Odyssey blocking buffer (927-40000; Li-Cor) in PBS, followed by ON incubation at 4 °C with the primary antibodies anti-calnexin (1:1000; ab22595; Abcam), anti-TSG101 (1:1000; NBP1-80659; Novus Biologicals) or anti-CD81 (1:1000; 10037; Cell Signalling Technology) in 1:4 diluted Odyssey blocking buffer in PBS. After a washing step, the membrane was incubated for 1 h at RT with the secondary antibody goat anti-rabbit DyLight 800 in 1:4 diluted Odyssey blocking buffer in PBS supplemented with 0.01% SDS and 0.1% Tween-20. After washing the membrane, bands were visualized using the Odyssey Fc Imager (Li-Cor, Germany).

### Mass spectrometry and label free protein quantification

For proteomics of qEV enriched EVs separated from conditioned medium of the apical side of mouse primary CPE cells stimulated with AβO or scrambled peptide, qEV fractions 2 and 3 were collected and pooled as described above. Next, they were vacuum evaporated and lysed in RIPA buffer (150 mM NaCl, 50 mM Tris pH 8.0, 1% NP-40, 0.5 mM EDTA pH 8.0, 0.5% sodium deoxycholate, cOmplete protease inhibitor cocktail (Sigma)). Samples were reduced in 20 mM dithiothreitol (DTT) (Sigma) at 56 °C for 2 h and subsequently alkylated in 40 mM iodoacetamide (IAA) (Sigma) for 30 min in the dark at RT. After alkylation, samples were transferred to a 10 kDa centrifugal spin filter and sequentially washed with 300 μl of 8 M urea three times and with 300 μl of 20 mM ammonium bicarbonate two times by centrifugation at 14,000*g*. Tryptic digestion was performed by adding trypsin (1:100; Promega) in 200 μl of 20 mM ammonium bicarbonate at 37 °C for 16 h. Peptides were recovered in the supernatant upon centrifugation at 14,000*g*. The filter was rinsed twice with 100 μl of 20 mM ammonium bicarbonate to increase peptide recovery. ZipTip (84850; Thermo Scientific) purification was performed and the peptides were eluted in 100 µl of the elution solvent. Equal amounts of peptides (1 µg) of each sample in 10 µl elution solvent where used for LC–MS/MS analysis, which was performed using an Ultimate 3000 RSLC nano LC (Thermo Scientific) in‐line connected to a LTQ Velos Orbitrap mass spectrometer (Thermo Scientific). Peptides were first trapped on a trapping column (made in‐house, 100 μm internal diameter (I.D.) × 20 mm, 5 μm C18 Reprosil‐HD beads (Dr. Maisch, Ammerbuch‐Entringen, Germany)). Subsequently, peptides were loaded onto an analytical column (made in‐house, 75 μm I.D. × 150 mm, 3 μm C18 Reprosil‐HD beads (Dr. Maisch)) which was packed in the nanospray needle (PicoFrit SELF/P PicoTip emitter, PF360‐75‐15‐N‐5, New Objective, Woburn, MA, USA). Peptides were initially loaded using 0.1% TFA in water/acetonitrile, 2/98 (v/v) and separated using a linear gradient from 98% of solvent A’ (0.1% formic acid in water/acetonitrile, 2/98 (v/v)) to 55% of solvent B’ (0.1% formic acid in water/acetonitrile, 20/80 (v/v)) for 150 min at a flow rate of 300 nl/min. This was followed by a 5 min wash reaching 97% of solvent B’. The mass spectrometer was operated in data‐dependent, positive ionization mode, automatically switching between MS and MS/MS acquisition for the 20 most abundant peaks in a given MS spectrum.

### Proteome data analysis

The mass spectrometry proteomics data have been deposited in the ProteomeXchange Consortium via the PRIDE partner repository with the dataset identifier PXD021665. MaxQuant [62] (version 1.5.7.4) was used to identify the generated MS/MS spectra using default settings. Here, oxidation of methionine and acetylation of protein N‐termini were used as variable modifications and carbamidomethylation of cysteine as a fixed modification. Further, trypsin was set as the used protease with two missed cleavages allowed. The peptide mass tolerance was set at 20 ppm for the first search and 4.5 ppm for the main search. The mass tolerance on fragment ions was set to 20 ppm. Spectra were searched against the mouse section of the Swiss-Prot database (16,859 protein entries). For label-free quantification (LFQ), MaxLFQ embedded in MaxQuant (version 1.5.7.4) was performed on proteins identified with at least two razor or unique peptides. The LFQ intensity values obtained by MaxLFQ were used for data analysis in the Perseus environment (version 1.5.3.2). Here, the intensity values were log2-transformed and filtered for reverse identifications. Proteins with at least two peptides identified were withheld for further quantification. Missing values were replaced with the help of the imputation function of Perseus based on a normal distribution of LFQ intensities (width 0.3 and standard deviation of 1.8). Multiple scatter plots with Pearson correlation were used to assess for correlation between the samples within a given group and between groups. Protein, peptide and site false discovery rates (FDR) were set to 0.01. Ingenuity pathway analysis software (IPA; Ingenuity Systems Inc., Redwood City, CA, USA) was used to perform the enrichment of canonical pathways. The Database for Annotation, Visualization and Integrated Discovery (DAVID) knowledgebase (version 6.8) was used for gene ontology (GO) term enrichment analysis. Proteins identified in the CPE primary cell EV proteome (scrambled and AβO combined, only proteins detected in at least two out of three replicates were taken into account) were submitted as the “query list” and the *Mus musculus* proteome was used as background list.

### Aβ ELISA

To determine the Aβ_1–42_ levels in the CSF of APP/PS1 mice, we performed Aβ ELISA as we described previously [14]. In brief, 96-well Maxisorp Nunc Immunoplates (430314; Thermo Scientific) were coated ON at 4 °C on a shaker with 50 µl anti-Aβ_1–42_ antibody (1.5 μg/ml; JRF/cAb42/26) in coating buffer (10 mM Tris–HCl, 10 mM NaCl, 10 mM NaN_3_ in 500 ml distilled H_2_O, pH 8.5) per well. Plates were washed five times with PBST (PBS + 0.05% Tween-20), after which residual protein binding sites were blocked for 4 h at RT with 100 µl blocking buffer (0.1% casein buffer). 30 µl of either Aβ_1–42_ standard (A-1163-1; rPeptide) or CSF (1:100 in sterile PBS) was mixed with 30 µl detection antibody (JRF/AbN/25 coupled to HRPO (Janssen Pharmaceutica); 1:2000 in blocking buffer). After blocking, ELISA plates were washed five times with PBST and 50 µl of the standard/sample-detection mixtures was added to the ELISA plates. Plates were incubated ON at 4 °C, while slowly shaking. Absorption at 450 nm was measured after adding 50 µl of TMB substrate solution (555214; BD Biosciences OptEIA™), followed by stopping buffer (50 µl 1 M H_2_SO_4_). The amount of Aβ_1–42_ was determined with GraphPad Prism 8.3 using the nonlinear regression model.

### Novel object recognition test

Cognitive behavior was investigated using the novel object recognition (NOR) test, as previously described [14, 63]. This NOR test is based on the innate tendency of mice to preferentially explore novel objects over familiar ones. The test consists of three phases: habituation, training and testing*.* The mice were brought into the testing room 30 min before the experiment to familiarize with the surroundings. For the habituation phase, mice were placed in a clear, acrylic box (40 cm × 40 cm × 40 cm) and allowed to explore the arena without objects for 5 min. The training phase was performed one day after the habituation phase. During the training trial, two identical objects were placed at two opposite positions within the box at the same distance from the nearest corner. The mice were allowed to explore the identical objects for 5 min after which they were returned to their home cage. In the testing phase, memory was tested either 15 min (short-term memory; STM) or 24 h (long-term memory; LTM) after the training phase. Mice were placed back in the same box for 5 min after replacing one of the familiar objects with a novel one. A different novel object was used in the STM compared to the LTM testing phase whereas the familiar objects remained unchanged along the training and testing phases. Exploration of the objects by the mice was defined as touching the object with their nose or mouth. All trials were videotaped and scored manually. The percentage preference for the novel object was calculated using the following formula: percentage novel object preference = (novel object exploration time/(novel object exploration time + familiar object exploration time)) × 100%. A value > 50% indicates preference for the novel object. To exclude the existence of olfactory cues, the box and the objects were thoroughly cleaned with 20% ethanol after each trial.

### Statistics

Statistics was performed using GraphPad Prism 8.3 (Graphpad Software, Inc.). Data are presented as means ± standard error of mean (SEM). NTA, ExoView, ELISA and TEM data were analyzed with an unpaired t-test unless mentioned differently. Bio-Plex, qPCR and cognition data were analyzed with a one-way ANOVA unless mentioned differently. Significance levels are indicated on the graphs: *, 0.01 ≤ *P* < 0.05; **, 0.001 ≤ *P* < 0.01; ***, 0.0001 ≤ *P* < 0.001; ****, *P* < 0.0001.

### EV-TRACK

All relevant data of our experiment were submitted to the EV-TRACK knowledgebase (EV-track ID: EV200066) [64].

## Results

### Transgenic APP/PS1 mice show elevated EV production in the CSF and CP tissue at early stages of disease

**Fig. 1:**
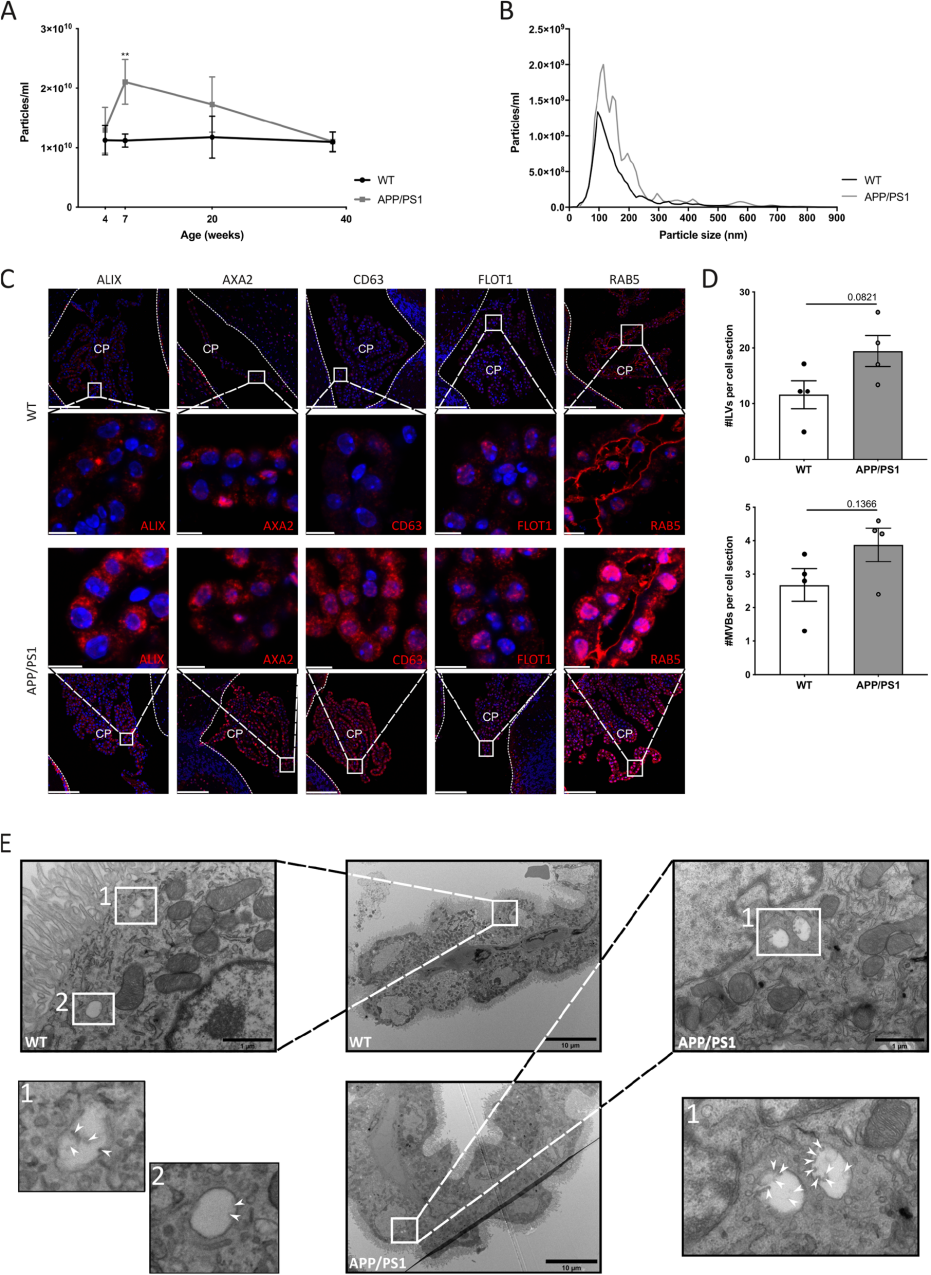
Analysis of extracellular vesicles (EVs) in cerebrospinal fluid (CSF) and choroid plexus (CP) of APP/PS1 mice. (**a**, **b**) nanoparticle Tracking Analysis (NTA; NanoSight) quantification (**a**) of CSF particles from 4 (n = 15 and n = 9), 7 (n = 20 and n = 13), 20 (n = 7 and n = 5) and 38 (n = 8 and n = 17) weeks old wild-type (WT) (black) and APP/PS1 (grey) mice. Size distribution (**b**) of CSF particles from 7 weeks old WT (black) (n = 20) and APP/PS1 (grey) (n = 13) mice. (**c**) Representative confocal images of ALIX, AnnexinA2 (ANXA2), CD63, Flotillin1 (FLOT1) and RAB5 (red) in the CP of 7 weeks old WT and APP/PS1 mice (n = 3). Nuclei are counterstained with Hoechst (blue) and the ependymal cell layer lining the ventricle wall is indicated by the white line. Scale bar represents 100 µm in the overview images and 10 µm in the zoomed-in images. (**d**, **e**) Quantification (**d**) and representative transmission electron microscopy (TEM) images (**e**) of 10 weeks old WT (black) and APP/PS1 (grey) mice (n = 4). Amount of multivesicular bodies (MVBs) per cell section and amount of intraluminal vesicles (ILVs) per cell section are displayed. Two regions of 20 CPE cells were manually counted for each biological replicate, resulting in the average amount of MVBs and ILVs in 40 CPE cells per cell section. White arrow heads point to ILVs present in MVBs. Scale bar represents 10 µm in the overview images and 1 µm in the zoomed-in images

CSF of transgenic APP/PS1 mice was analyzed using NTA (NanoSight). Early during disease progression (7 weeks of age), we observed higher levels of particles in APP/PS1 mice compared to age-matched non-transgenic littermates, while levels normalized at later stages (Fig. 1a, b, Additional file 1: Figure S1A–D). To determine whether these particles could be EVs produced by the CP, similar to what we described in sepsis [56], we analyzed the expression of several EV markers, namely ALIX, ANXA2, CD63, FLOT1 and RAB5, in brain samples from early stage AD mice compared to WT age-matched control mice. This revealed that several tested EV markers were strongly induced in the CP of APP/PS1 compared to age-matched WT mice (Fig. 1c). Moreover, in control mice, RAB5 was mainly localized close to the basal side of the CP cells while in APP/PS1 mice the signal was also clearly present at the apical side.

Size distribution analysis of the particles (Fig. 1b) shows that the detected CSF particles are in the size range of EVs, including exosomes. Exosomes are derived from MVBs, i.e. late endosomes that contain ILVs in their luminal compartment. MVBs can either be degraded in lysosomes or can fuse with plasma membrane and release ILVs into the extracellular space as exosomes [65]. TEM analysis of the CP of early stage AD mice was used to study the presence of MVBs and ILVs. As shown in Fig. 1d, although not significant, quantification showed more MVBs and ILVs in APP/PS1 mice compared to age-matched controls. Representative TEM images are displayed in Fig. 1e**.** Together, these data suggest that at least part of the particles present in the CSF of young APP/PS1 mice derive from the CP.

### Icv injection of AβO induces increased secretion of EVs into the CSF

**Fig. 2:**
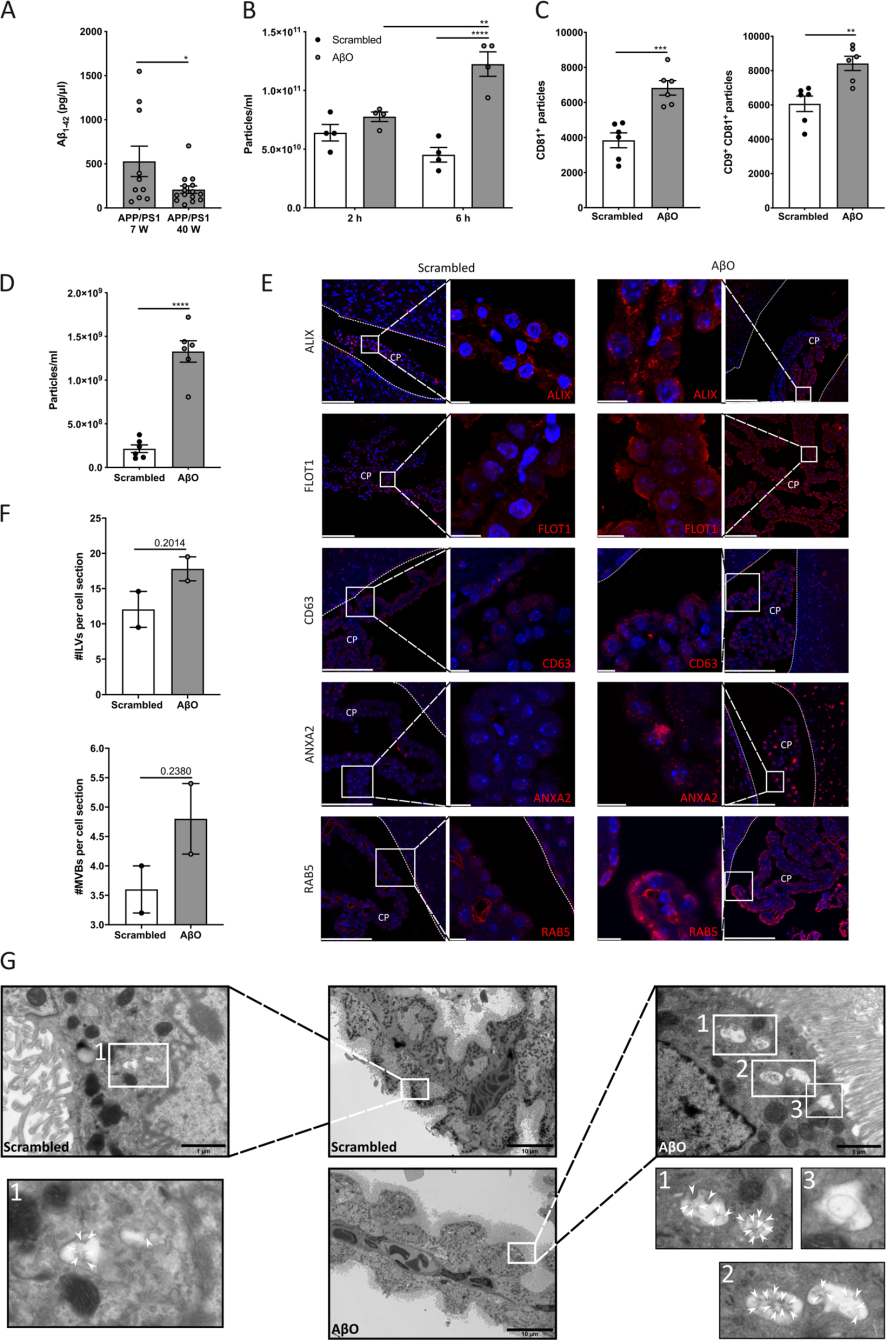
Analysis of extracellular vesicle (EV) secretion after intracerebroventricular (icv) injection of Aβ oligomers (AβO). (**a**) ELISA analysis of soluble Aβ_1–42_ levels in the cerebrospinal fluid (CSF) of 7 (n = 10) and 40 (n = 15) weeks old APP/PS1 mice. (**b**) Nanoparticle Tracking Analysis (NTA; NanoSight) quantification of the amount of particles in CSF 2 and 6 h after icv injection of scrambled peptide (black) or AβO (grey) in C57BL/6J mice (n = 4), analyzed using 2-way ANOVA. (**c**) ExoView analysis of the amount of CD81 captured—CD81 positive and CD9 captured—CD81 positive EVs in CSF 6 h after icv injection of scrambled peptide (black) or AβO (grey) in C57BL/6J mice (n = 6). For each biological replicate, the presented result is the average from three different technical replicates on the chip. (**d**) NTA (NanoSight) quantification of the particles in the medium of primary CPE cells after 2 h of stimulation with scrambled peptide (black) or AβO (grey) and 24 h of incubation (n = 6). (**e**) Representative confocal images of ALIX, AnnexinA2 (ANXA2), CD63, Flotillin1 (FLOT1) and RAB5 (red) in the CP 6 h after the icv injection of scrambled peptide or AβO in C57BL/6J mice (n = 3). Cell nuclei are counterstained with Hoechst (blue). The ependymal cells that line the ventricle are indicated by a white line. Scale bar represents 100 µm in the overview images and 10 µm in the zoomed-in images. (**f**, **g**) Quantification (**f**) and representative transmission electron microscopy (TEM) images (**g**) of CP tissue, isolated from mice 4 h after the icv injection with scrambled peptide or AβO in C57BL/6J mice (n = 2). Amount of multivesicular bodies (MVBs) per cell section and amount of intraluminal vesicles (ILVs) per cell section are displayed. Two regions of 20 CPE cells were manually counted for each biological replicate, resulting in the average amount of MVBs and ILVs in 40 CPE cells per cell section. White arrow heads point to ILVs present in MVBs. Scale bar represents 10 µm in the overview images and 1 µm in the zoomed-in images

The CSF Aβ levels of APP/PS1 transgenic mice are known to peak at the age of 6 weeks, and gradually decline to minimum levels by the age of 72 weeks [66, 67]. Indeed, we observed significantly higher soluble Aβ_1–42_ levels in young APP/PS1 mice compared to older APP/PS1 mice (Fig. 2a). This led us to investigate whether the CSF-presence of Aβ_1–42_ and especially AβO, the most neurotoxic Aβ species, could be responsible for the elevated EV-levels in the CSF of young APP/PS1 mice. Therefore, we prepared AβO as described before [4, 14, 58, 59], and injected them into the left lateral brain ventricle of young C57BL/6J mice, followed by CSF NTA (NanoSight) analysis 2 and 6 h later. As presented in Fig. 2b, we observed a significant increase in particles in the CSF 6 h after icv injection of AβO. Because with the NTA technique all particles within a similar size range are measured, we performed an additional ExoView analysis on the CSF samples. This revealed an increase of the EV-associated tetraspanins CD9 and CD81 (Fig. 2c) in CSF of AβO injected mice, indicating that the increase in AβO-induced particle secretion into the CSF as measured by NTA is at least in part caused by an AβO-induced increase in the number of CD9 and CD81 positive EVs in the CSF.

Next, to study whether the CP is involved in EV production, primary CPE cultures were prepared and cultured on transwell inserts. Confluent CPE cells were treated with AβO added to the apical compartment for 2 h. After stimulation, cells were washed with HBSS, fresh Opti-MEM was added and conditioned medium was collected after 24 h of incubation. Similar to the in vivo observations, a significant increase in amount of particles was observed in the supernatant of AβO stimulated primary CPE cultures (Fig. 2d), suggesting that the CPE cells produce EVs upon stimulation with AβO in vitro and in vivo. Immunostainings for several EV markers, namely ALIX, ANXA2, CD63, FLOT1 and RAB5 revealed a strong induction of all tested EV markers in the CP of AβO injected mouse brain samples (Fig. 2e). Moreover, upon AβO, similar to the APP/PS1 mice, RAB5 was localized close to the apical side and displayed a punctuated pattern. In contrast, signals from CD63, FLOT1, ALIX and ANXA2 show a more cytoplasmic, but also punctuated pattern. This suggests that the signals arise from MVBs, from which exosomes originate. Again, this was further studied using TEM analysis of CP tissue of mice that were icv injected with AβO or scrambled control. This confirmed the presence of more ILVs and MVBs in AβO injected mice, although this was not significant (Fig. 2f). Representative TEM images are displayed in Fig. 2g. Together, these data identify the CP as at least a partial source of the AβO-induced EV release into the CSF.

Additionally, we used the ExoView technology to assess the presence of a CP specific marker on CSF-EVs. To this end, we made use of TTR, a carrier protein involved in the regulation of thyroid hormone concentration in extracellular fluids of which the brain levels are almost exclusively derived from the CP [68, 69]. As represented in Additional file 1: Figure S2A, we could show that TTR is present in at least part of the CSF-EVs which suggests that the CP is an important source of the EVs that are present in the CSF. Indeed, TTR is also clearly detected in EVs separated from the secretome of CP explants (Additional file 1: Figure S2B) and primary CPE cells (Additional file 1: Figure S2C), two setups in which the analysed secretome is exclusively derived from the CP.

### AβO-induced EV secretion has an inflammatory effect on brain cells in vitro

**Fig. 3:**
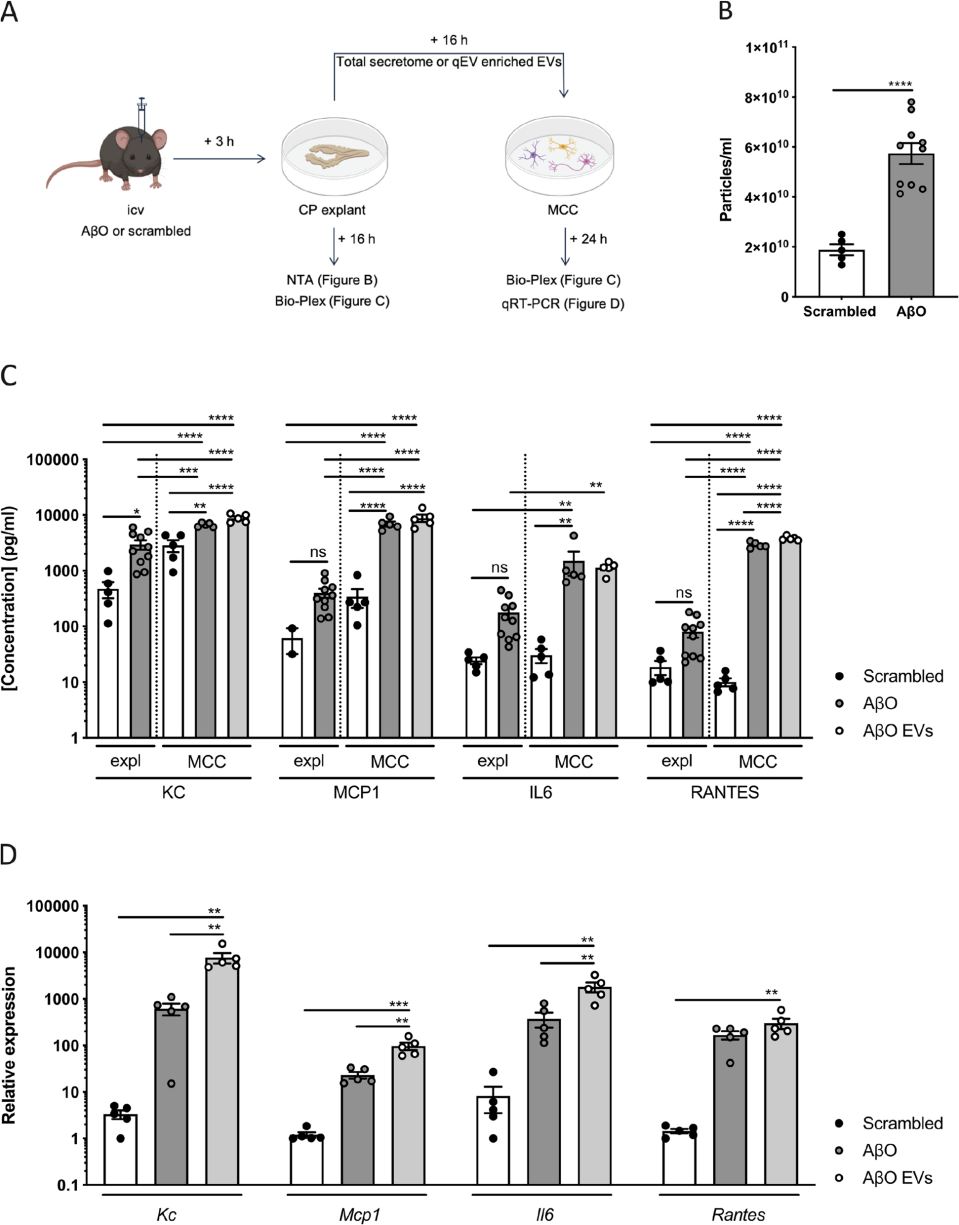
Effect of extracellular vesicle (EV) secretion by the choroid plexus (CP) after intracerebroventricular (icv) injection of Aβ oligomers (AβO) on mixed cortical cultures (MCC). (**a**) Overview of the experimental setup. The Figure was partially created with BioRender. (**b**) Nanoparticle Tracking Analysis (NTA; NanoSight) quantification of the amount of particles in medium of CP explants that were isolated from C57BL/6J mice mice 3 h after icv injection of scrambled peptide (black) or AβO (grey) (n = 5 and n = 10) and cultured for 16 h in Opti-MEM. (**c**, **d**) Cytokine and chemokine analysis of CP explant and MCC supernatant (**c**) or MCC cells (**d**). CP explants were isolated from C57BL/6J mice mice 3 h after icv injection of scrambled peptide (black) or AβO (grey) (n = 5 and n = 10) and cultured for 16 h in Opti-MEM, after which the supernatant was collected for Bio-Plex analysis. MCC were incubated with the complete secretome of CP explants derived from scrambled peptide (black) or AβO (grey) injected mice or incubated with qEV enriched EVs separated from the secretome of CP explants derived from AβO (white) injected mice. 24 h after incubation the supernatant and cells were collected and analyzed using respectively Bio-Plex assay for KC, MCP1, IL6 and RANTES and qRT-PCR analysis for *Kc*, *Mcp1*, *Il6* and *Rantes* (n = 5)

To investigate the effect of CP-derived EVs on target cells, we prepared CP explants from C57BL/6J mice icv injected with either scrambled peptide or AβO. Three hours after injection, CP was isolated and incubated in vitro for 16 h in Opti-MEM. Analysis of the secretome of the different explants using NTA (NanoSight) resulted in similar results as the CSF analysis: the presence of AβO induces an increase in particle secretion by the CP (Fig. 3b). Cytokine and chemokine levels were analyzed using Bio-Plex assay and this revealed a significant increase of KC, MCP1, IL6 and RANTES in the secretome of CP explants coming from mice injected with AβO compared to the scrambled condition, indicating a pro-inflammatory response induced by AβO (Fig. 3c). For TNF and IL1β, no significant differences were detected (Additional file 1: Figure S3A). Next, we transferred the total CP secretome or qEV enriched EVs separated from the CP explant supernatant to MCC to study the effect of the CP-derived secretome and EVs on target brain cells. According to established criteria [70], the quality of these EV fractions was verified using different methods. NTA (ZetaView) showed that the highest concentration of particles within the size range of EVs eluted in fractions 2 and 3, whereas the highest protein concentrations were obtained in fractions 14–15 (Additional file 1: Figure S4A, B). TEM confirmed the presence of typical cup-shaped EVs in pooled qEV fractions 2 and 3 (Additional file 1: Figure S4C, D). Furthermore, ExoView analysis showed that these EVs are positive for the tetraspanins CD9 and CD81 (Additional file 1: Figure S4E). The presence of the tetraspanin CD81 and the cytosolic protein TSG101 in qEV fraction 2 + 3, but not in fraction 13 + 14, was shown via western blot (Additional file 1: Figure S4F). Finally, the purity of the EVs was confirmed by the largely reduced presence of the endoplasmic reticulum marker calnexin in qEV fraction 2 + 3 in comparison with the cell lysate (Additional file 1: Figure S4F). Twenty-four hours later, conditioned medium and MCC cells were isolated and analyzed for cytokine and chemokine expression using respectively Bio-Plex assay and qRT-PCR analysis. As presented in Fig. 3c, d, several cytokines and chemokines, namely KC, MCP1, IL6 and RANTES, were significantly higher in medium of MCC treated with total CP secretome from mice injected with AβO compared to the scrambled condition, whereas also the gene expression of *Kc*, *Mcp1* and *Il6* was significantly upregulated in the MCC cells. Strikingly, the inflammatory response was equally high or even significantly higher in medium and MCC cells treated with EVs separated from AβO-injected mice CP explant secretome, indicating that the inflammatory effect is mediated by the EVs and suggesting that the AβO-induced EVs have a pro-inflammatory effect on their target cells.

**Fig. 4:**
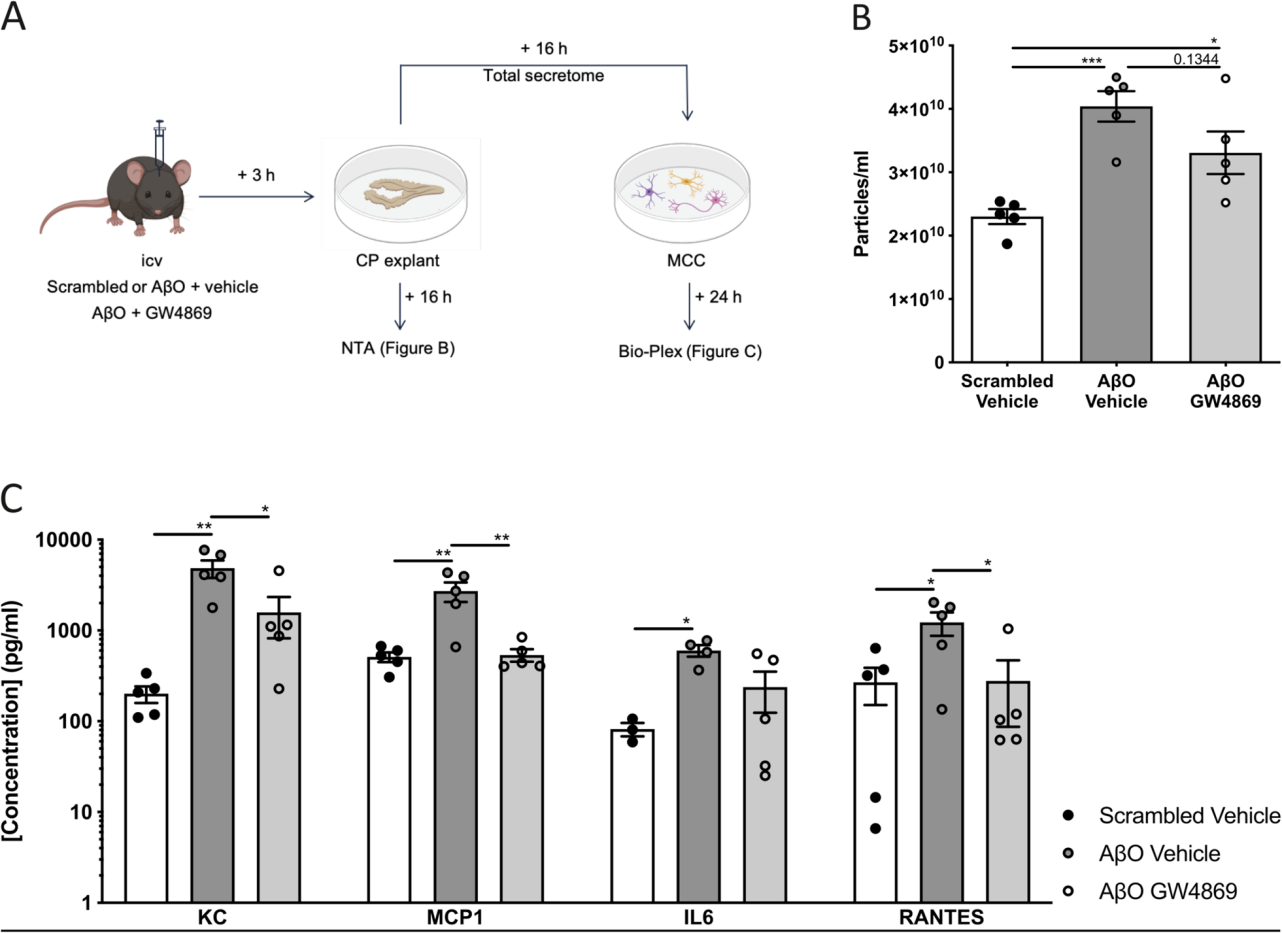
Effect of inhibiting exosome secretion by the choroid plexus (CP) after intracerebroventricular (icv) injection of Aβ oligomers (AβO) on mixed cortical cultures (MCC). (**a**) Overview of the experimental setup. The Figure was partially created with BioRender. (**b**) Nanoparticle Tracking Analysis (NTA; NanoSight) quantification of the amount of particles in medium of CP explants that were isolated from C57BL/6J mice mice 3 h after icv injection of scrambled peptide + vehicle (black), AβO + vehicle (grey) or AβO + GW4869 (white) (n = 5) and cultured for 16 h in Opti-MEM. Data were analyzed using 1-way Anova (**c**) Cytokine and chemokine analysis of MCC supernatant. MCC were incubated with the complete secretome of CP explants derived from scrambled peptide + vehicle (black), AβO + vehicle (grey) or AβO + GW4869 (white) injected mice. 24 h after incubation the supernatant was collected and analyzed using Bio-Plex assay for KC, MCP1, IL6 and RANTES (n = 5)

Next, we wanted to investigate whether inhibiting the AβO-induced EV secretion would abrogate this pro-inflammatory effect. Therefore, we made use of the pharmaceutical compound GW4869 which is a specific, non-competitive inhibitor of nSMase 2 used to study the effect of blocking exosome biogenesis and release [71, 72]. Three hours after the icv injection of mice with scrambled peptide, AβO or AβO together with GW4869, CP was isolated and incubated in vitro for 16 h in Opti-MEM. Analysis of the secretome of the different explants using NTA (NanoSight) revealed that the co-injection of AβO with GW4869 can reduce the AβO-induced EV secretion by the CP (Fig. 4b). Importantly, the GW4869 effect is less pronounced in this ex vivo experiment compared to the in vivo data. This is expected, since GW4869 was not present during the 16 h explant culturing period to avoid transfer of GW4869 to the next step of the experiment. In this next step, we transferred the CP secretome to MCC to study the effect of EV inhibition on the target cells. Twenty-four hours later, conditioned medium was isolated and analyzed for cytokine and chemokine expression using Bio-Plex. As represented in Fig. 4c and Additional file 1: Figure S3B, KC, MCP1 and RANTES but not IL1β nor TNF were significantly lower in medium of MCC treated with secretome of CP explants coming from mice co-injected with AβO and GW4869 compared with the AβO condition. This again suggests that the AβO-induced EVs have a pro-inflammatory effect on their target cells whereby inhibiting the AβO-induced EV secretion abrogates this effect.

### AβO-induced EVs are enriched for C3 and the icv injection of AβO induces C3 upregulation in the CP

**Fig. 5:**
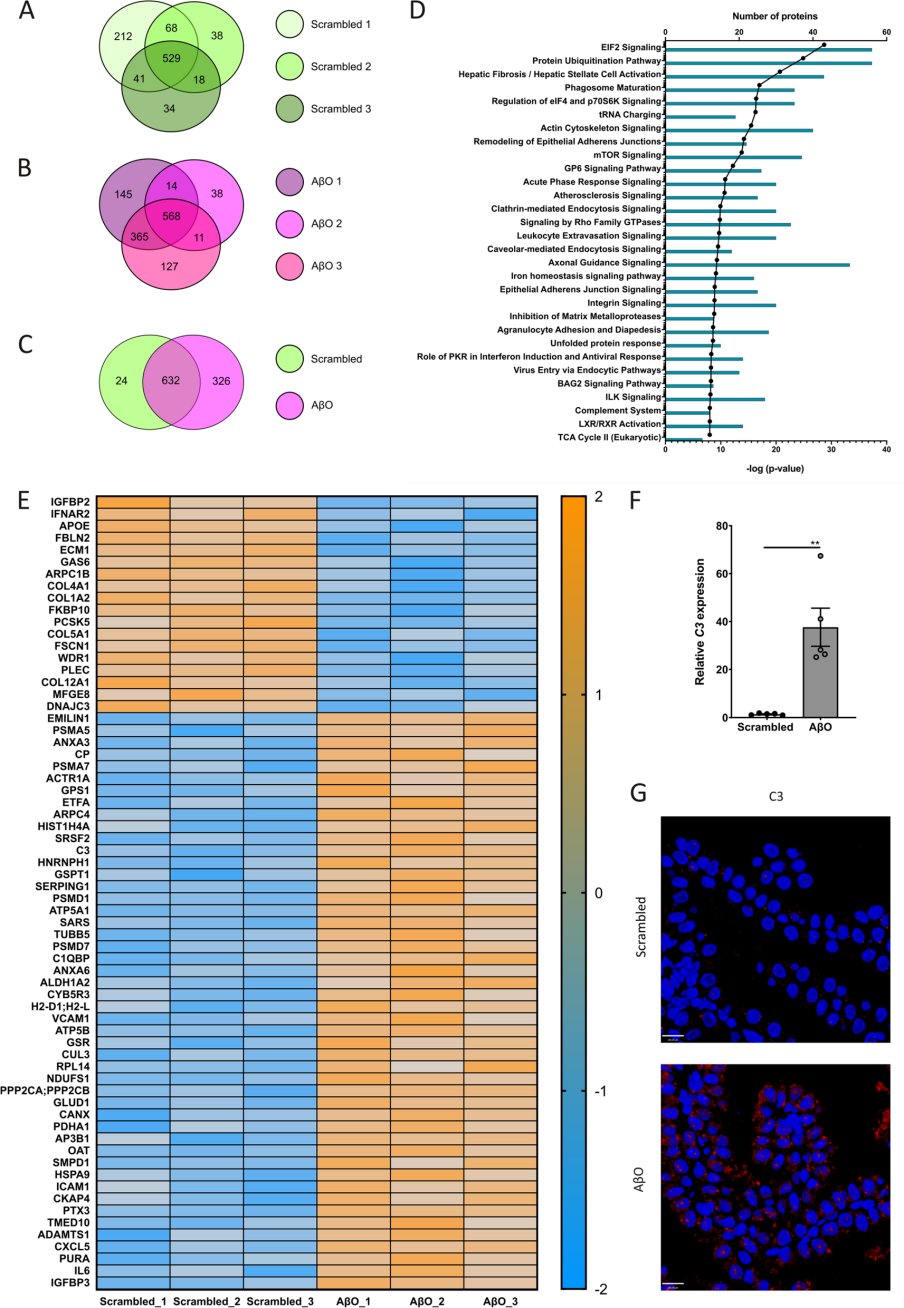
Proteome analysis of primary choroid plexus epithelial (CPE)-derived extracellular vesicles (EVs) and complement activation in the CP after stimulation with Aβ oligomers (AβO). (**a**, **b**) Venn diagrams showing overlap of the total number of proteins identified in EVs separated from the apical medium of primary CPE cells after 2 h of stimulation with (**a**) scrambled peptide (green) or (**b**) AβO (pink) (n = 3). (**c**) Venn diagram showing overlap of proteins identified in at least two out of three replicates of EVs separated from the apical medium of primary CPE cells after 2 h of stimulation with scrambled peptide (green) or AβO (pink) and 24 h of incubation (n = 3). (**d**) Ingenuity pathway analysis (IPA) of differentially regulated proteins in EVs separated from the apical medium of primary CPE cells after 2 h of stimulation with AβO or scrambled peptide and 24 h of incubation (n = 3). (**e**) Heat map of z-scores (calculated from log_2_-transformed label-free quantification (LFQ) protein intensities) for proteins differentially expressed (*P* < 0.01) in EVs separated from the apical medium of primary CPE cells after 2 h of stimulation with AβO or scrambled peptide and 24 h of incubation (n = 3). Proteins are ranked in ascending order according to their fold change value for the AβO versus the scrambled group. (**f**) qRT-PCR analysis of the complement component 3 (*C3*) in the CP 6 h after the icv injection of scrambled peptide (black) or AβO (grey) in C57BL/6J mice (n = 5), analyzed using an unpaired t-test. (**g**) 3D reconstructions of representative confocal images of C3 (red) in the CP 6 h after the icv injection of AβO or scrambled peptide in C57BL/6J mice (n = 3). Cell nuclei are counterstained with Hoechst (blue). Scale bar represents 100 µm

We performed an LFQ proteome analysis on EVs separated from the apical medium of primary CPE cells that were stimulated with AβO or scrambled peptide. The EVs were separated from the medium using qEV columns and subjected to proteome analysis after pooling qEV fractions 2 and 3. Successful enrichment of EVs in these fractions was verified according to established criteria [70]. Similar to the CP explant separated EVs, NTA (NanoSight) analysis showed that the highest concentration of particles within the size range of EVs eluted in fractions 2 and 3, whereas the highest protein concentration was obtained in fraction 13 (Additional file 1: Figure S5A, B). TEM confirmed the presence of typical cup-shaped EVs in pooled fractions 2 and 3 (Additional file 1: Figure S5C, D), which are positive for the tetraspanins CD9 and CD81 according to ExoView analysis (Additional file 1: Figure S5F). Furthermore, western blot analysis revealed the presence of CD81 and TSG101 in qEV fractions 2 + 3 but not in qEV fractions 13 + 14 (Additional file 1: Figure S5E). Additionally, the endoplasmic reticulum marker calnexin was identified in cell lysate but not in the EV fraction, confirming the purity of the separated EVs (Additional file 1: Figure S5E). We identified 1092, 631 and 1071 proteins in the three AβO biological replicates (Fig. 5a) and 850, 653 and 622 proteins in the three scrambled biological replicates (Fig. 5b). Respectively 75.6% and 69.8% of the identified proteins were present in at least two out of three biological replicates. Reproducibility of the proteomics was high, reflected by the Pearson correlation coefficients of the comparison of the LFQ intensities of the different samples that vary between 0.856 and 0.961 (Additional file 1: Figure S6A). Comparison of the proteomes of EVs from AβO or scrambled peptide stimulated primary CPE cells, taking into account only proteins detected in at least two out of three replicates, revealed that 24 and 326 proteins were specific for respectively the scrambled peptide and AβO stimulated primary CPE cells (Fig. 5c). This indicates that AβO stimulation strongly alters the composition of the EV proteome. Comparing the CPE primary cell EV proteome (scrambled and AβO combined, only taking into account proteins detected in at least two out of three replicates) with the *Mus musculus* proteome list available on the Vesiclepedia website [73] (version 4.1) showed that 84.5% of the EV proteome overlaps with the Vesiclepedia list (Additional file 1: Figure S5G, H). Although this result has to be interpreted with caution because the Vesiclepedia database contains information about EVs derived from different biofluids using a variety of separation methods that differ in specificity [74], it does suggest that we enriched for EVs. To identify the pathways enriched in the proteome of EV samples, we performed a GO enrichment analysis using DAVID (version 6.8) [75]. We submitted the CPE primary cell EV proteome (scrambled and AβO combined, only taking into account proteins detected in at least two out of three replicates,) and used the *Mus musculus* proteome as the background list. As shown in Additional file 1: Figure S5I, the “extracellular exosome” pathway is the top enriched pathway in the submitted EV proteome, again proving successful EV purification. Other enriched GO-terms related to EVs include extracellular matrix, extracellular space and extracellular region. Next, we analyzed differentially expressed proteins by uploading all quantified proteins with corresponding fold change and *P* value in the IPA software. Figure 5d shows the top 30 canonical signaling pathways, including acute phase response signaling, leukocyte extravasation signaling and complement system, which clearly shows that the EV proteome is pro-inflammatory. Interestingly, the top pathway is eukaryotic elongation factor 2 (eIF2) signaling of which aberrant signaling has been implicated in the pathogenesis of AD [76]. Additionally, the presence of the inhibition of MMPs pathway is an interesting observation since we have previously shown the involvement of MMPs at the blood–CSF barrier in response to AβO [4]. To look deeper into the most differentially expressed proteins (*P* < 0.01) upon AβO stimulation, we calculated the z-scores from the log_2_-transformed LFQ protein intensities and plotted these values in a heat map (Fig. 5e). Proteins are ranked in ascending order according to their fold change value for the AβO versus the scrambled group. The exact fold change values are depicted in the Volcano plot in Additional file 1: Figure S6B. Several interesting proteins previously shown to be linked to AD, such as insulin-like growth factor-binding protein 3 (IGFBP3), intracellular adhesion molecule 1 (ICAM1) and sphingomyelin phospodiesterase 1 (SMPD1) are upregulated in the AβO condition. Of particular interest, also the complement protein C3 is significantly upregulated in the EVs separated from the medium of AβO-stimulated primary CPE cells compared to the scrambled stimulated cells. Because compelling evidence indicates that the complement pathway plays an important role in AD [43], we investigated the effect of AβO on the expression of *C3* in the CP. As shown in Fig. 5f, the *C3* gene expression was significantly induced in the CP upon the icv injection of AβO. In agreement with this, we observed a strong induction of this complement protein in the CP of AβO injected mice using immunostaining (Fig. 5g).

### Inhibition of exosome secretion prevents acute AβO-induced cognitive decline

**Fig. 6:**
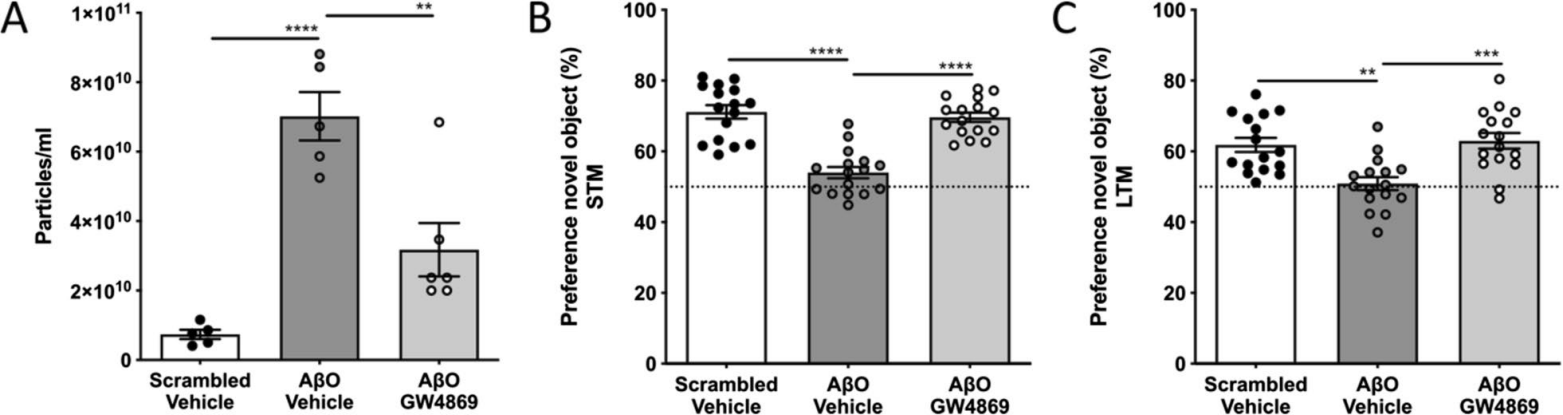
Effect of inhibiting exosome secretion after intracerebroventricular (icv) injection of Aβ oligomers (AβO) on short-term memory (STM) and long-term memory (LTM) (**a**) Nanoparticle Tracking Analysis (NTA; NanoSight) quantification of the amount of particles in the cerebrospinal fluid (CSF) 6 h after icv injection of scrambled peptide + vehicle (black), AβO + vehicle (grey) or AβO + GW4869 (white) in C57BL/6J mice (n = 5–6). (**b**) Analysis of the STM in C57BL/6J mice 24 h after injection of either scrambled peptide + vehicle (black), AβO + vehicle (grey) or AβO + GW4869 (white) (n = 16; pooled data of two independent experiments). (**c**) Analysis of the LTM in C57BL/6J mice 48 h after injection of either scrambled peptide + vehicle (black), AβO + vehicle (grey) or AβO + GW4869 (white) (n = 16; pooled data of two independent experiments)

The pharmaceutical compound GW4869 is a specific, non-competitive inhibitor of nSMase 2 that is used to study the effect of blocking exosome biogenesis and release [71, 72]. We performed icv injection of scrambled peptide, AβO or AβO together with GW4869 and analyzed the CSF 6 h later using NTA (NanoSight). This revealed that the icv injection of AβO alone induced a significant increase in particles into the CSF which was reduced by co-injection of AβO with GW4869 (Fig. 6a), similarly as we have shown in the context of systemic inflammation [56]. One of the main symptoms of AD is cognitive decline, and soluble forms of AβO were shown to be involved in inducing these cognitive impairments [77–79]. Based on the observed pro-inflammatory effects of CP-derived AβO-induced EVs on MCC and the pro-inflammatory content of primary CPE-derived AβO-induced EVs, we analyzed whether exosome inhibition using GW4869 could prevent acute AβO-induced cognitive decline. Mice were subjected to the NOR test and both STM and LTM were analyzed. As expected, scrambled peptide injected control mice show a novel object preference in the STM trial of ~ 70%, which indicates sustained memory. In contrast, AβO injected mice showed a novel object preference of only ~ 54%, which indicates impaired memory function (Fig. 6b). Interestingly, co-injection of AβO along with GW4869 prevented the acute AβO-induced memory decline and resulted in a memory performance similar to the control group, as shown by the novel object preference of ~ 70% (Fig. 6b). In the LTM trial, the same pattern was observed (Fig. 6c). These results show that blocking AβO-induced EV secretion into the CSF is able to prevent the acute AβO-induced loss of cognitive decline, indicating an important role for EVs in the pathogenesis of AD.

## Discussion

EVs are nanosized, double membrane vesicles produced by almost every cell type including prokaryotes. Several recent studies indicate that EVs play a role in neurodegenerative diseases such as AD. EVs produced by brain parenchymal cells such as microglia, astrocytes and neurons have been a primary focus in AD, while the CP that is responsible for the CSF production has not yet been investigated. We previously showed that the CP responds to peripheral inflammatory triggers by releasing EVs into the CSF, thereby spreading inflammation to the CNS [56]. In this study, we investigated the role of CP-derived EVs in two in vivo mouse models for AD. Although no single AD model recapitulates all aspects of the disease spectrum, the selected models allowed us to specifically study the amyloidosis component of AD. As a first model, we used APP/PS1 transgenic mice that harbor human transgenes for both the APP bearing the Swedish mutation (KM670/671NL) and PSEN1 bearing an L166P mutation, both under the control of the neuron-specific Thy1 promotor [57]. Here, we show that the EV levels in the CSF from these APP/PS1 transgenic mice are increased compared to WT counterparts early on in the disease. In contrast, no difference could be observed at late stages, indicating an early pathogenic role of these EVs in AD. Moreover, close examination of EV markers in the CP of the APP/PS1 mice clearly revealed a strong induction of several tested EV markers at early stages of AD, thus correlating with their CSF profile. Interestingly, it is known that APP/PS1 transgenic mice show increased levels of Aβ40 and Aβ42 in their brain and CSF starting from a young age, with Aβ42 levels exceeding Aβ40 several-fold [57, 66, 67]. Also, in our study, we found increased soluble Aβ_1–42_ levels in the CSF of young APP/PS1 mice, suggesting that the increased levels of CSF Aβ_1–42_ might be responsible for the EV production by the CP.

To investigate this hypothesis further, we made use of a second AD model, being the icv injection of AβO in C57BL/6J mice. Hereby, AβO are injected directly into the CSF via the left lateral ventricle which makes this model perfectly suited to address our research question, namely whether the presence of AβO in the CSF can induce EV secretion. Soluble AβO are an intermediate stage between monomeric Aβ and senile plaques, responsible for mediating immunological changes in the brain [80, 81]. We and others have shown that this model recapitulates several aspects of AD, including memory decline [14, 79, 82–85]. In agreement with the results from APP/PS1 mice, injection of AβO also induced a profound increase in EV secretion into the CSF by the CP. This was again confirmed by immunofluorescence analysis of EV markers in CP samples of AβO injected mice compared to scrambled peptide injected controls. Additional experiments performed in vitro and ex vivo clearly demonstrated that the CP responds to AβO and secretes excessive EVs into the medium, signifying that the presence of AβO in the CSF is sufficient to induce EV secretion by the CP. Interestingly, we were able to inhibit EV production in vivo using GW4869, indicating that the produced EVs are indeed derived from MVBs, and are at least in part exosomes. However, we cannot exclude the possibility of the presence of other vesicle types, nor that a part of the detected EVs in the CSF might originate from other brain cells. In this context, it has been shown that human CSF-EVs were enriched for several brain-specific proteins, although CP associated proteins were predominant [86]. Adding to this, we could show that at least part of the CSF-EVs are positive for the CP specific marker TTR similarly to what we showed previously [56], suggesting that the CP is an important source of CSF-EVs.

Additionally, functional studies using secretome derived from in vivo AβO stimulated CP explants clearly demonstrate that the CP secretome has a pro-inflammatory effect on target cells, being MCC. Strikingly, when only EVs separated from the CP secretome were transferred to the MCC, an equal or even increased pro-inflammatory response was observed. This might be explained by the fact that when we are only transferring EVs separated from the CP secretome, all other components of the secretome including anti-inflammatory cytokines are not transferred. Consequently, this might lead to an overall higher inflammatory response in the target cells. In conclusion, our data indicate that AβO-induced, CP-derived EVs are responsible for the observed pro-inflammatory effect. Vice versa, reducing the AβO-induced EV secretion by the CP using GW4869 abrogated the pro-inflammatory effect on the target cells. Importantly, neuroinflammation plays a fundamental role in the aggravation of neurodegenerative diseases, including AD. Misfolded proteins and protein/peptide aggregates bind to cell surface receptors such as pattern recognition receptors (PRRs) of glial cells and initiate an innate immune response in the CNS, which is characterized by the release of pro-inflammatory mediators and acceleration of disease progression and severity. Moreover, several studies pointed out the fact that neuroinflammation precedes the appearance of pathological hallmarks of AD such as senile Aβ plaques and neurofibrillary tangles [87–93]. It was shown that both AβO and P-tau are responsible for provoking an innate immune response in the brain which includes activation of pro-inflammatory cytokines and chemokines which further leads to the inflammation in the CNS [94, 95]. Additionally, we have previously shown that AβO induce inflammation in the CP and leakage of the blood–CSF barrier by activating MMPs and TNFR1 signaling [4, 14]. The results presented here suggest that also CPE-derived EVs are an important contributor to the inflammatory component of AD, which we investigated further by focusing on the content of CPE-derived EVs.

It is known that EVs contain several types of molecules, including proteins, lipids and genetic material, which can affect recipient cells [17]. Proteome analysis of EVs separated from the apical medium of primary CPE cells that were stimulated with AβO or scrambled peptide revealed that 326 proteins were specific for the EV proteome after AβO treatment. Pathway analysis on the differentially expressed proteins showed that several inflammatory pathways including acute phase response signaling, leukocyte extravasation signaling and the complement system were upregulated, clearly showing that the EV proteome is pro-inflammatory. Interestingly, the top pathway was eIF2 signaling of which aberrant signaling has been implicated in the pathogenesis of AD [76]. Genetic reduction of the eIF2 kinase improved memory deficits in Tg19959 and APP/PS1 mice, without altering Aβ pathology [96]. Plotting the z-scores calculated from the log_2_-transformed LFQ protein intensities from the most differentially expressed proteins (*P* < 0.01) in a heat map showed that several interesting proteins related to AD were upregulated in EVs separated from the AβO condition. For instance, the protein with the highest fold change for the AβO versus the scrambled condition was IGFBP3. Interestingly, IGFBP3 is increased in the temporal cortex of human AD patients [97]. Additionally, Aβ stimulation of primary astrocyte cultures resulted in release of IGFBP3 that could induce tau phosphorylation in neurons [97]. Another protein that stands out is ICAM-1 of which increased levels are present in the CSF of preclinical, prodromal and dementia AD stages [98]. Also, SMPD1, otherwise known as acid sphingomyelinase (ASM), is upregulated in the AβO condition. It was shown that in 9 month old APP/PS1 mice, ASM brain and plasma levels are increased whereas partial genetic depletion or pharmacological inhibition of ASM in these mice reduced Aβ deposition and improved memory function [99]. As a mechanism, it was proposed that increased ASM levels lead to defective autophagy due to impaired lysosomal biogenesis, resulting in an inability to break down appropriate substrates during the autophagy process [99]. Collectively, our proteome analysis revealed that the content of the AβO-induced CPE-derived EVs might affect several pathological processes related to AD.

In our opinion, one of the most interesting upregulated proteins in the AβO-induced EVs was C3 as compelling evidence points towards an important role for the complement pathway in AD. Most studies indicate glial cells as a source of complement in the AD brain [43], whereas only limited data have been obtained about the CP. In our study, we used a combination of in vivo, ex vivo and in vitro approaches to investigate EV secretion by the CP in response to AβO. We cannot exclude the contribution of for example CP-infiltrating immune cells to the observed effects in the in vivo and the ex vivo approach, but importantly, the in vitro approach that was applied for the proteomics experiment allowed us to specifically investigate the role of the CPE cells themselves. This signifies the importance of the CPE cells in the generation of C3-containing EVs in response to AβO. Various reports already showed that several complement components localized at the CP in other disorders. The complement proteins C1q and C3 have been detected in the CP in systemic hypertension [100], hepatosplenic schistosomiasis [101] and liver cirrhosis [102]. Also, in the CP of multiple sclerosis patients as well as in other neurological conditions and non-neurological controls, C1q and C3b were present [103]. Additionally, levels of several complement regulators (CD35, CD46, CD55 and CD59) were shown in the CP of patients who suffered from meningococcal meningitis [104]. Interestingly, also in the case of AD patients, the presence of C1q in the CP was shown [105]. More recently, complement activation in the CP of AD patients was reported by the presence of C1q-ApoE complexes, C3 and C5. Strikingly, this even correlated with cognitive decline [106]. However, to the best of our knowledge, we are the first to show that icv injected AβO directly upregulate the expression of C3 in the CPE cells, both on mRNA and protein level. These data indicate a direct link between AβO and complement activation at the blood–CSF barrier. Interestingly, it has been suggested that complement is able to influence the number of circulating vesicles [107]. However, in our study further research will be required to determine whether the AβO-induced increase in C3 in the CP and the AβO-induced EV secretion by the CP might be linked. Additionally, further studies will be needed to determine which part of the AβO-induced effects can be attributed to the presence of C3 inside the AβO-induced EVs.

Knowing that the CP-derived EVs have a pro-inflammatory content and are able to induce a pro-inflammatory response in brain cells in vitro, we next studied whether inhibition of the EV secretion might have beneficial effects in our AD model. Therefore, we made use of GW4869, an inhibitor of nSMase 2. Inhibition of nSMase 2, resulting in blockage of EV formation and subsequent release from MVBs, resulted in reduced tau propagation in a mouse model of rapid tau propagation [38]. Similarly, genetic deletion of nSMase 2 in 5XFAD mice ameliorated AD pathology in this mouse model [37]. Also, AβO administration into the brain results in an acute impairment of cognitive function [79, 82–85]. Previously, we showed that icv injection of AβO resulted in short-term and long-term memory deficits in the NOR test [14]. Strikingly, here we revealed that administration of AβO along with GW4869 abolished acute AβO-induced memory deficits, indicating that the inhibition of EV secretion can prevent the occurrence of acute AβO-induced memory deficits in mice.

## Conclusions

**Fig. 7:**
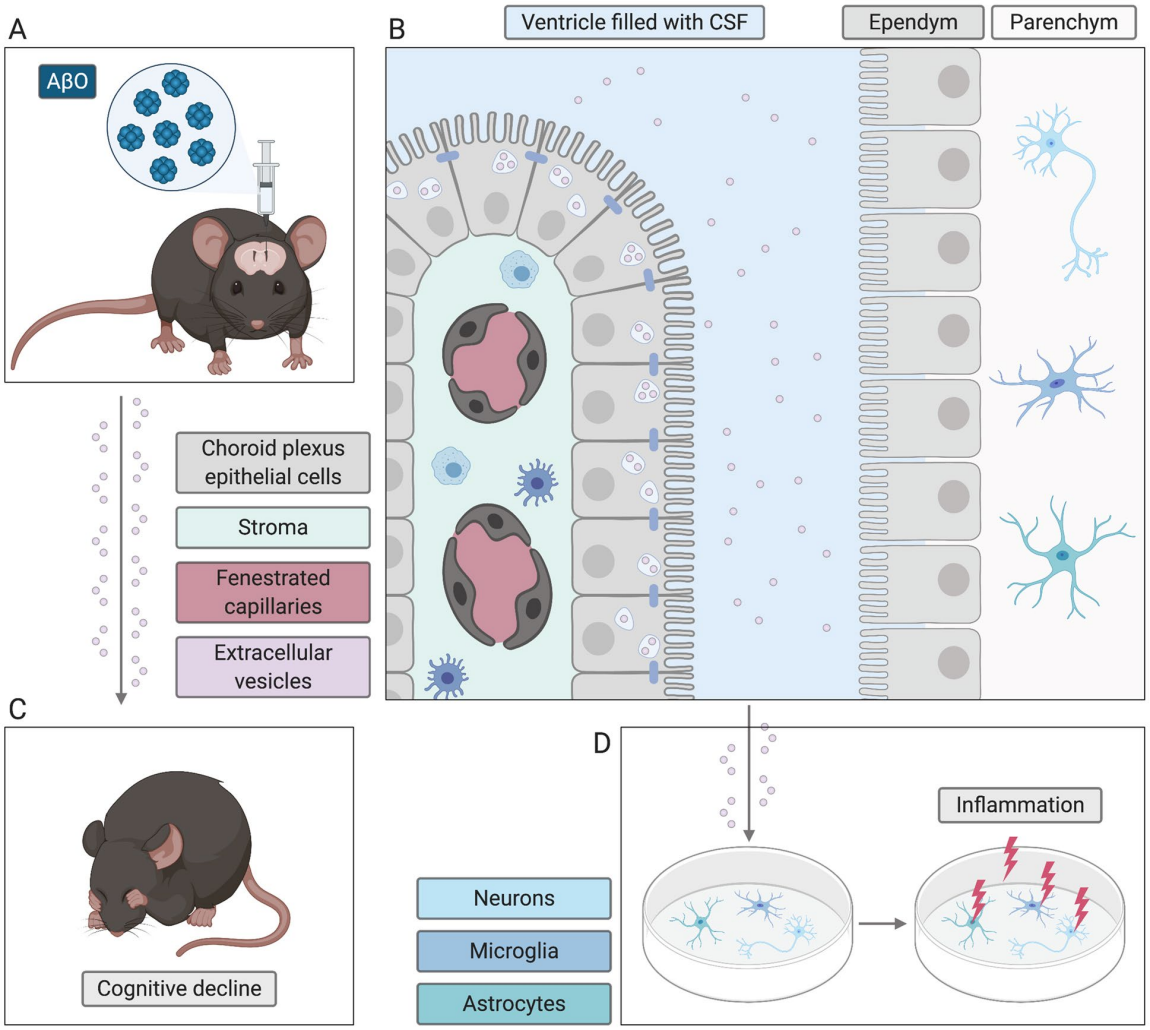
As a mouse model for Alzheimer’s disease (AD), we injected amyloid beta oligomers (AβO) into the cerebrospinal fluid (CSF) of wild-type (WT) mice via their left lateral brain ventricle (*i.e.* intracerebroventricular (icv) injection) (**Panel A**). This resulted in an increased release of extracellular vesicles (EVs) into the CSF, whereby these EVs are at least in part derived from the choroid plexus (CP) (**Panel B**). Interestingly, AβO-induced, CP-derived EVs carry several pro-inflammatory proteins including the complement protein C3. Furthermore, we could show that the AβO-induced, CP-derived EVs exert a pro-inflammatory response on brain target cells in vitro (**panel D**). Strikingly, these EVs also play a role in loss of cognitive function (**panel C**), since blocking the EV secretion using GW4869 protected against the AβO-induced cognitive decline. Image created with BioRender

In summary (Fig. 7), we demonstrated that AβO induce the release of EVs into the CSF by the CP. These AβO-induced, CP-derived EVs contain several pro-inflammatory proteins, including C3, and induce a pro-inflammatory effect on their target cells in the brain. Strikingly, inhibition of exosome secretion using GW4869 prevented acute AβO-induced loss of memory. Our results indicate that AβO-induced, CP-derived and C3-containing EVs exert detrimental effects in AD pathology. This suggests that inhibition of EV production by the CP might be an interesting therapeutic approach to be explored in the prevention or treatment of AD.

## Supplementary Information

**Additional file 1.** Supplementary Figures S1-S6 and Appendix Table S1.

## Data Availability

The mass spectrometry proteomics data are available via ProteomeXchange with identifier PXD021665.

## Abbreviations

AD: Alzheimer’s disease
ANXA2: Annexin A2
APP: Amyloid precursor protein
ASM: Acid sphingomyelinase
AβO: Amyloid beta oligomers
BBB: Blood–brain barrier
C1q: Complement component 1q
C3: Complement component 3
CNS: Central nervous system
CP: Choroid plexus
CPE: Choroid plexus epithelial
CSF: Cerebrospinal fluid
DAVID: Database for Annotation, Visualization and Integrated Discovery
DTT: Dithiothreitol
eIF2: Eukaryotic elongation factor 2
EVs: Extracellular vesicles
FDR: False discovery rates
FLOT1: Flotillin1
GO: Gene ontology
HFIP: Hexafluoroisopropanol
IAA: Iodoacetamide
ICAM1: Intracellular adhesion molecule 1
Icv: Intracerebroventricular
IGFBP3: Insulin-like growth factor-binding protein 3
ILVs: Intraluminal vesicles
IPA: Ingenuity pathway analysis
LFQ: Label-free quantification
LTM: Long-term memory
MCC: Mixed cortical cultures
MCI: Mild cognitive impairment
MMPs: Matrix metalloproteinases
MVBs: Multivesicular bodies
NEAA: Non-essential amino acids
NOR: Novel object recognition
nSMase 2: Neutral sphingomyelinase 2
NTA: Nanoparticle Tracking Analysis
ON: Overnight
P-tau: Hyperphosphorylated tau
PFA: Paraformaldehyde
PRR: Pattern recognition receptor
PSEN1: Presenilin 1
RT: Room temperature
SEM: Standard error of mean
SMPD1: Sphingomyelin phospodiesterase 1
SPF: Specific pathogen-free
STM: Short-term memory
TEM: Transmission electron microscopy
TNF: Tumor necrosis factor
TNFR1: Tumor necrosis factor receptor 1
TTR: Transthyretin
WT: Wild-type
