# Supplementary material for: Importance of extracellular vesicle secretion at the blood–cerebrospinal fluid interface in the pathogenesis of Alzheimer’s disease
Source: Acta Neuropathol Commun. 2021 Aug 23;9:143. doi: 10.1186/s40478-021-01245-z (PMC8381545; doi:10.1186/s40478-021-01245-z)
Supplement: Supplementary file 1 — Additional file 1. Supplementary Figures S1-S6 and Appendix Table S1. [file 40478_2021_1245_MOESM1_ESM.docx]

**Supplementary Figure S1**


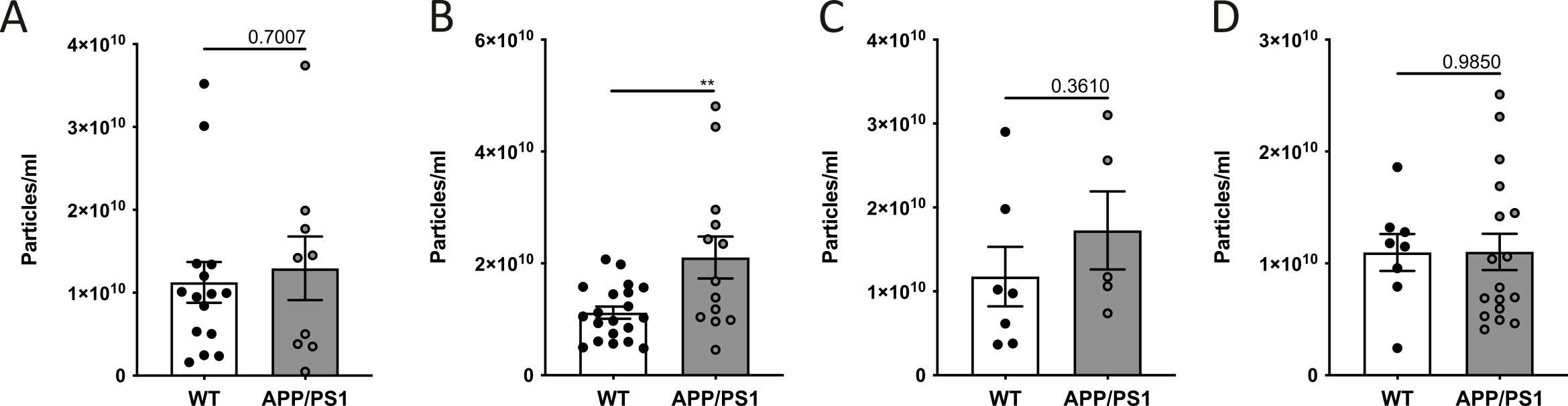


**Supplementary Figure 1. Analysis of particles in cerebrospinal fluid (CSF) of APP/PS1 mice. (A-D)** Nanoparticle Tracking Analysis (NTA; NanoSight) quantification of CSF particles from (A) 4 (n=15 and n=9), (B) 7 (n=20 and n=13), (C) 20 (n=7 and n=5) and (D) 38 (n=8 and n=17) weeks old wild-type (WT) (black) and APP/PS1 (grey) mice.

**Supplementary Figure S2**

**
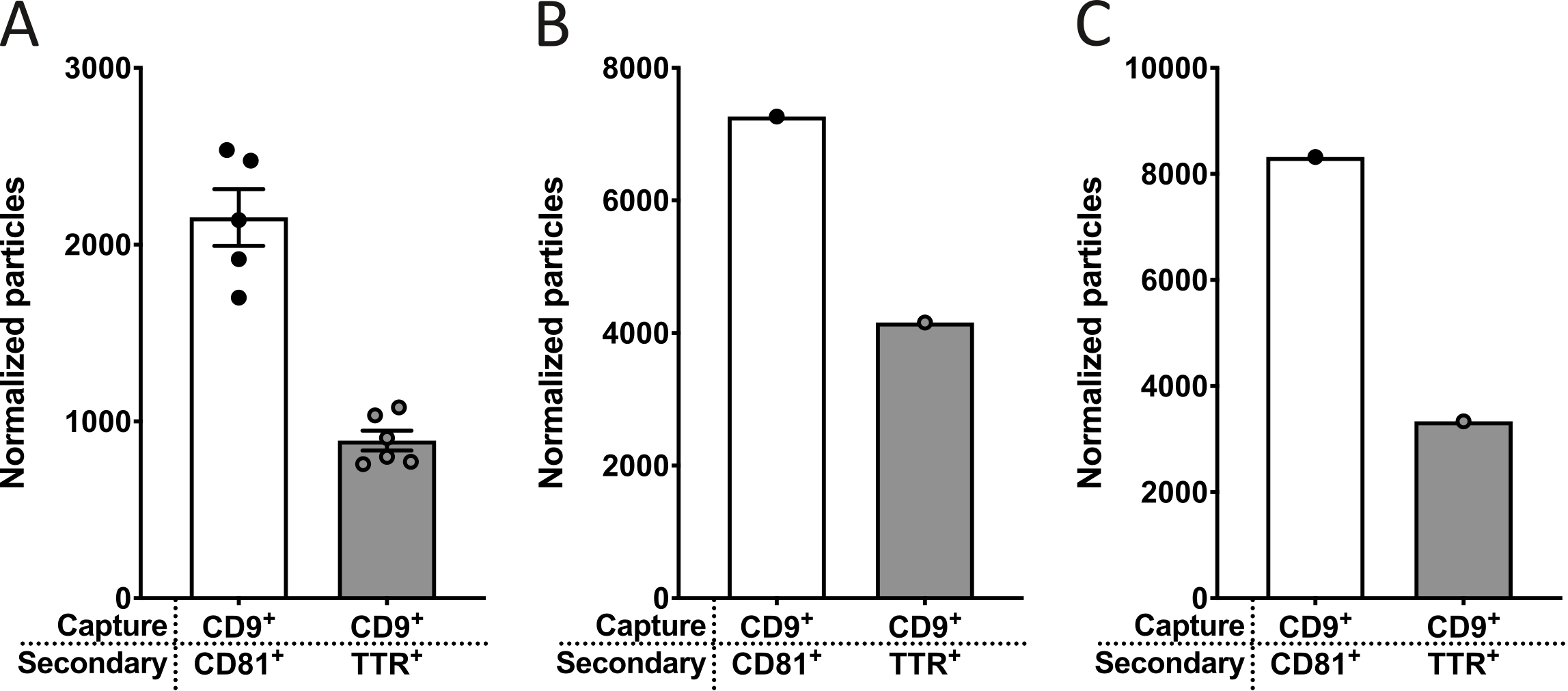
**

**Supplementary Figure 2. Analysis of the presence of transthyretin (TTR) on extracellular vesicles (EVs) in cerebrospinal fluid (CSF) and medium of choroid plexus (CP) explants and primary choroid plexus epithelial (CPE) cells.** (**A-C**) ExoView analysis of the amount of CD9 captured - CD81 positive (black) and CD9 captured - TTR positive (grey) particles in (A) CSF (B) EVs separated from medium of CP explant cultures and (C) EVs separated from medium of primary CPE cells. For each biological replicate, the presented result is the average from three different technical replicates on the chip.

**Supplementary Figure S3**


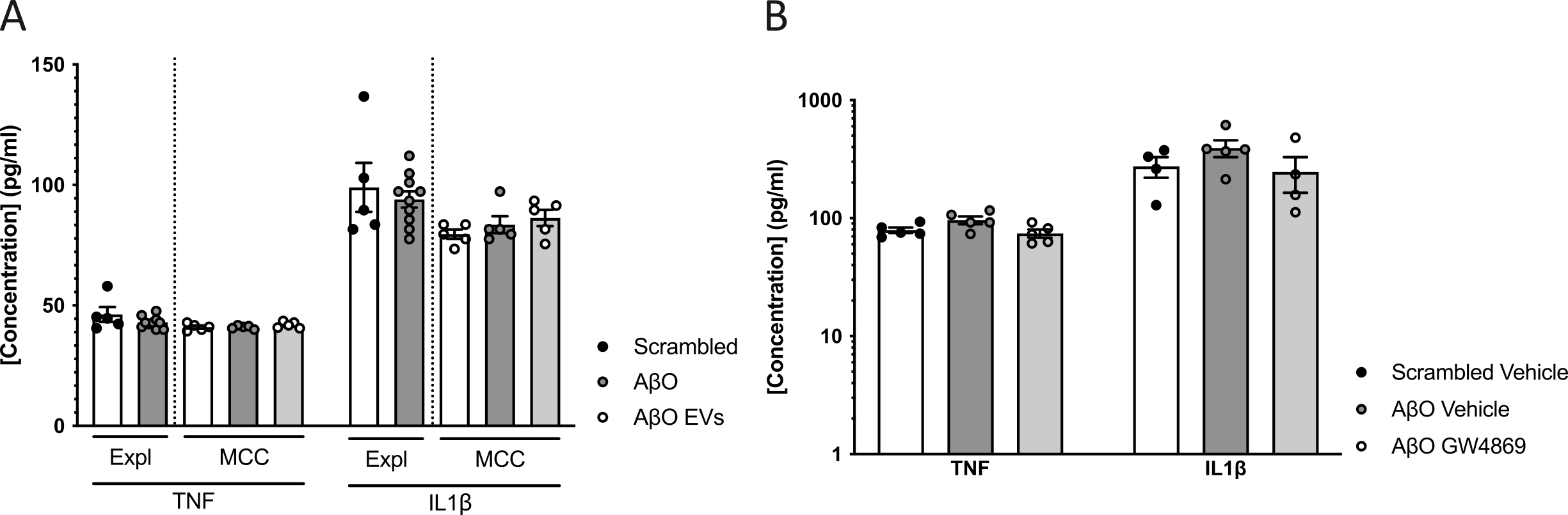


**Supplementary Figure 3. Effect of extracellular vesicle (EV) secretion by the choroid plexus (CP) after intracerebroventricular (icv) injection of Aβ oligomers (AβO) on mixed cortical cultures (MCC). (A)** Cytokine and chemokine analysis of CP explant and MCC supernatant. CP explants were isolated from C57BL/6J mice mice 3 h after icv injection of scrambled peptide (black) or AβO (grey) (n=5 and n=10) and cultured for 16 h in Opti-MEM, after which the supernatant was collected for Bio-Plex analysis. MCC were incubated with the complete secretome of CP explants derived from scrambled peptide (black) or AβO (grey) injected mice or incubated with qEV enriched EVs separated from the secretome of CP explants derived from AβO (white) injected mice. 24 h after incubation the supernatant was collected and analyzed using Bio-Plex assay for TNF and IL1β. **(B)** Cytokine and chemokine analysis of MCC supernatant. MCC were incubated with the complete secretome of CP explants derived from scrambled peptide + vehicle (black), AβO + vehicle (grey) or AβO + GW4869 (white) injected mice. 24 h after incubation the supernatant was collected and analyzed using Bio-Plex assay for TNF and IL1β (n=5).

**Supplementary Figure S4**


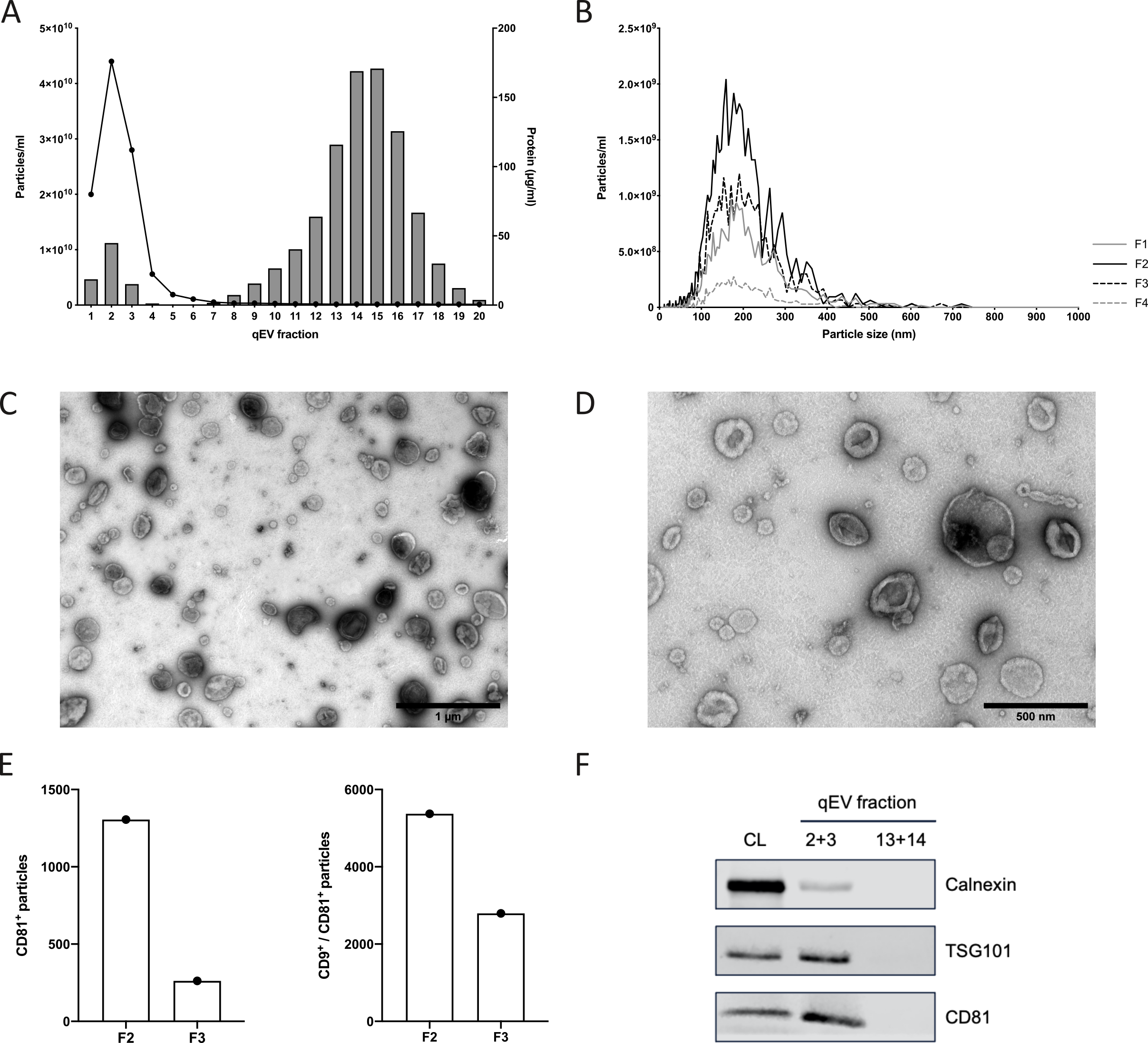


**Supplementary Figure 4. qEV-characterization of extracellular vesicles (EVs) separated from choroid plexus (CP) explant secretome. (A)** Quantification using Nanoparticle Tracking Analysis (NTA; ZetaView) (black interconnected dots) and protein concentration using Micro BCA (grey bars) of qEV enriched EV fractions 1-20 separated from the medium of CP explant cultures. **(B)** Size distribution profile of qEV enriched EV fractions 1-4 separated from the medium of CP explant cultures. **(C-D)** Transmission electron microscopy (TEM) images of qEV enriched and pooled fractions 2 and 3 separated from the medium of CP explant cultures. Scale bar represents 1 µm (C) and 500 nm (D). **(E)** ExoView analysis of the amount of CD81 captured - CD81 positive and CD9 captured - CD81 positive EVs in fractions 2 and 3 enriched from the medium of CP explant cultures. For both samples, the presented result is the average from three different technical replicates on the chip. **(F)** Western blot analysis for Calnexin, TSG101 and CD81 of cell lysate (CL) and qEV enriched and pooled EV fraction 2 + 3 and 13 + 14 separated from the medium of CP explants. 8 µg of protein was loaded on the gel.

**Supplementary Figure S5**


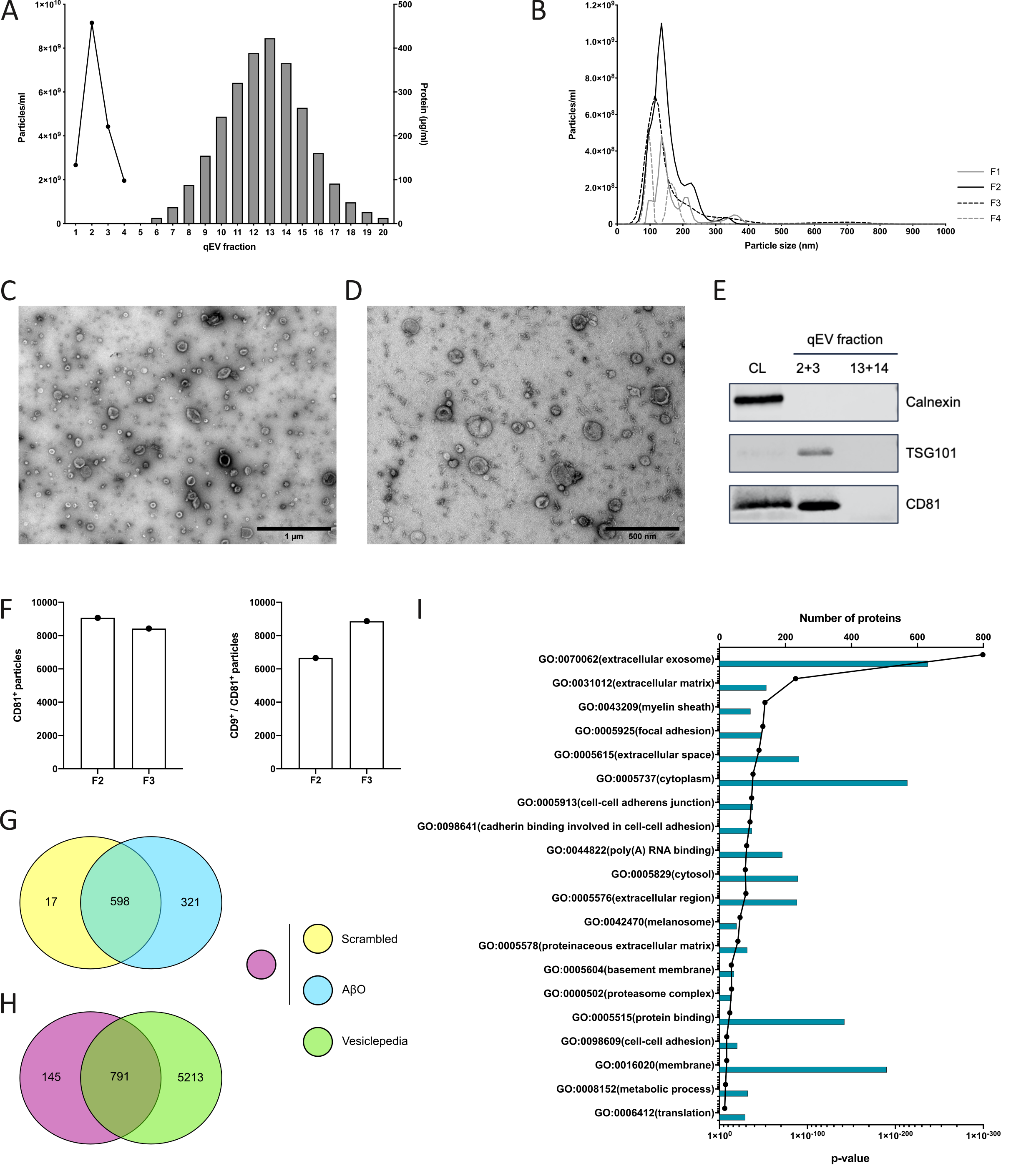


**Supplementary Figure 5. qEV-characterization of extracellular vesicles (EVs) separated from the medium of primary choroid plexus epithelial (CPE) cells. (A)** Quantification using Nanoparticle Tracking Analysis (NTA; NanoSight) (black interconnected dots) of qEV enriched EV fractions 1-4 and protein concentration using Micro BCA (grey bars) of qEV enriched EV fractions 1-20 separated from the medium of primary CPE cells. **(B)** Size distribution profile of qEV enriched EV fractions 1-4 separated from the medium of primary CPE cells. **(C-D)** Transmission electron microscopy (TEM) images of qEV enriched and pooled EV fraction 2 and fraction 3 separated from the medium of primary CPE cells. Scale bar represents 1 µm (C) and 500 nm (D). **(E)** Western blot analysis for Calnexin, TSG101 and CD81 of cell lysate (CL) and qEV enriched and pooled EV fraction 2 + 3 and 13 + 14 separated from the medium of primary CPE cells. 8 µg of protein was loaded on the gel. **(F)** ExoView analysis of the amount of CD81 captured - CD81 positive and CD9 captured - CD81 positive EVs in fractions 2 and 3 separated from the medium of primary CPE cells. For both samples, the presented result is the average from three different technical replicates on the chip. **(G)** Venn diagram showing overlap of proteins identified in qEV enriched EVs (fractions 2 and 3 pooled) separated from the apical medium of primary CPE cells after 2 h of stimulation with AβO (blue) or scrambled peptide (yellow) and 24 h of incubation (n=3). Only annotated proteins that were present in at least two out of three replicates were taken into account. **(H)** Venn diagrams showing overlap of the CPE primary cell EV proteome (scrambled and AβO; purple) with the *Mus musculus* proteome list available on the Vesiclepedia website (version 4.1; green). **(I)** Gene Ontology (GO) enrichment analysis using DAVID (version 6.8) of the CPE primary cell EV proteome (scrambled and AβO) compared with the *Mus musculus* proteome. Only proteins detected in at least two out of three replicates were taken into account. The blue bars represent the number of proteins and the black dots the p-values.

**Supplementary figure S6**

**
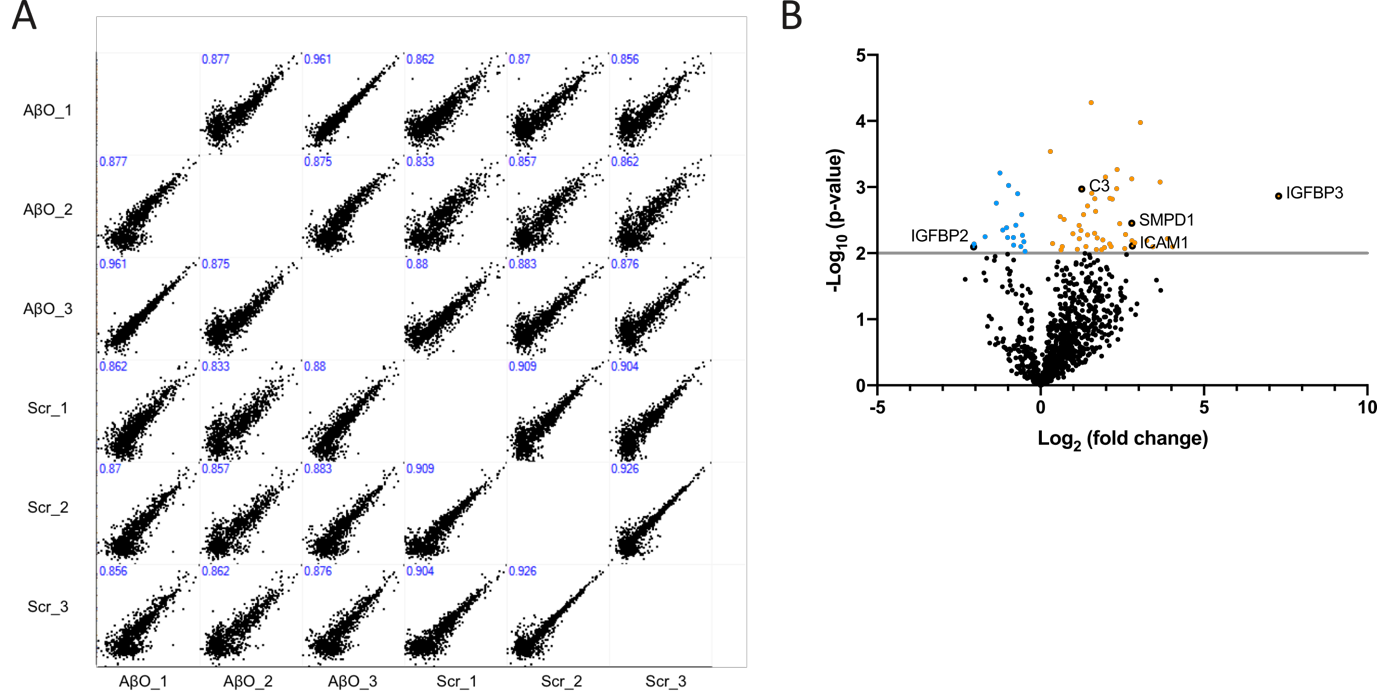
**

**Supplementary Figure 6. Proteome analysis of extracellular vesicles (EVs) separated from the apical medium of primary choroid plexus epithelial (CPE) cells after stimulation with Aβ oligomers (AβO). (A)** Scatter plots displaying label-free quantification (LFQ) intensities of the different proteome samples. The calculated correlation coefficient (Pearson correlation) for each pair of samples is indicated in blue at the left corner of the graphs. “AβO_1”, “AβO_2” and “AβO_3” are biological replicates derived from qEV enriched EVs from AβO-stimulated primary CPE cells. “Scr_1”, “Scr_2” and “Scr_3” are biological replicates derived from qEV enriched EVs from scrambled stimulated primary CPE cells. **(B)** Volcano plot of the p-values (Y-axis) versus the log_2_ fold change (X-axis) of all quantified proteins in EVs separated from the apical medium of primary CPE cells after 2 h of stimulation with AβO or scrambled peptide and 24 h of incubation (n=3). Blue indicates downregulated and orange indicates upregulated proteins (p < 0.01, represented by the horizontal line) in the AβO condition compared to the scrambled condition.

**Appendix Table S1**

| **Gene** | **Forward** | **Reverse** |
| --- | --- | --- |
| *Hprt* | AGTGTTGGATACAGGCCAGAC | CGTGATTCAAATCCCTGAAGT |
| *Rpl* | CCTGCTGCTCTCAAGGTT | TGGTTGTCACTGCCTCGTACTT |
| *Ubc* | AGGTCAAACAGGAAGACAGACGTA | TCACACCCAAGAACAAGCACA |
| *Kc* | GCTGGGATTCACCTCAAGAA | TCTCCGTTACTTGGGGACAC |
| *Mcp1* | TTAAAAACCTGGATCGGAACCAA | GCATTAGCTTCAGATTTACGGGT |
| *Il6* | TAGTCCTTCCTACCCCAATTTCC | TTGGTCCTTAGCCACTCCTTC |
| *Rantes* | GCTGCTTTGCCTACCTCTCC | TCGAGTGACAAACACGACTGC |
| *C3* | CCAGCTCCCCATTAGCTCTG | GCACTTGCCTCTTTAGGAAGTC |

**Appendix Table S1. Overview of the sequences of the forward and the reverse primers used for qPCR analysis.**
